# Criterion-Related Validity of the Distance- and Time-Based Walk/Run Field Tests for Estimating Cardiorespiratory Fitness: A Systematic Review and Meta-Analysis

**DOI:** 10.1371/journal.pone.0151671

**Published:** 2016-03-17

**Authors:** Daniel Mayorga-Vega, Raúl Bocanegra-Parrilla, Martha Ornelas, Jesús Viciana

**Affiliations:** 1 Department of Physical Education and Sport, University of Granada, Granada, Spain; 2 Faculty of Sciences of Physical Culture, Autonomous University of Chihuahua, Chihuahua, Mexico; Rutgers University -New Jersey Medical School, UNITED STATES

## Abstract

**Objectives:**

The main purpose of the present meta-analysis was to examine the criterion-related validity of the distance- and time-based walk/run tests for estimating cardiorespiratory fitness among apparently healthy children and adults.

**Materials and Methods:**

Relevant studies were searched from seven electronic bibliographic databases up to August 2015 and through other sources. The Hunter-Schmidt’s psychometric meta-analysis approach was conducted to estimate the population criterion-related validity of the following walk/run tests: 5,000 m, 3 miles, 2 miles, 3,000 m, 1.5 miles, 1 mile, 1,000 m, ½ mile, 600 m, 600 yd, ¼ mile, 15 min, 12 min, 9 min, and 6 min.

**Results:**

From the 123 included studies, a total of 200 correlation values were analyzed. The overall results showed that the criterion-related validity of the walk/run tests for estimating maximum oxygen uptake ranged from low to moderate (*r*_p_ = 0.42–0.79), with the 1.5 mile (*r*_p_ = 0.79, 0.73–0.85) and 12 min walk/run tests (*r*_p_ = 0.78, 0.72–0.83) having the higher criterion-related validity for distance- and time-based field tests, respectively. The present meta-analysis also showed that sex, age and maximum oxygen uptake level do not seem to affect the criterion-related validity of the walk/run tests.

**Conclusions:**

When the evaluation of an individual’s maximum oxygen uptake attained during a laboratory test is not feasible, the 1.5 mile and 12 min walk/run tests represent useful alternatives for estimating cardiorespiratory fitness. As in the assessment with any physical fitness field test, evaluators must be aware that the performance score of the walk/run field tests is simply an estimation and not a direct measure of cardiorespiratory fitness.

## Introduction

Physical fitness constitutes an integrated measure of all the functions and structures involved in the performance of physical activity [1]. Particularly cardiorespiratory fitness reflects the overall capacity of the cardiovascular and respiratory systems to supply oxygen during sustained physical activity [2]. Currently there is strong evidence that cardiorespiratory fitness constitutes an important predictor of morbidity and mortality [3]. Therefore, it is considered one of the most powerful markers of health, even above other traditional indicators such as weight status, blood pressure or cholesterol level [4].

Cardiorespiratory fitness testing may help to identify a target population for primary prevention and health promotion policies [5]. The maximal oxygen uptake (VO_2_max) attained during a laboratory and graded maximal exercise test is commonly considered the criterion measure [6]. Nevertheless, since the direct determination of VO_2_max by laboratory testing requires sophisticated and expensive equipment, qualified examiners, and long testing sessions, this technique is not feasible in several settings such as in sports clubs, physical education lessons, or large scale research studies [7]. In these settings, the performance score obtained during cardiorespiratory fitness field tests could be a useful alternative to estimate VO_2_max [7]. Since the early interest in physical fitness testing in the 1950-60s, many field tests have been proposed [8]. The walk/run field tests are probably the most widely used [8,9], but there is still no consensus regarding the most appropriate distance or time of the walk/run test for estimating cardiorespiratory fitness [10].

Each primary study about the criterion-related validity of the distance- and time-based walk/run tests only constitutes a single piece of evidence [11]. For example, when Cooper [12] studied the criterion-related validity of the 12 min walk/run test, he found a high correlation coefficient, however, later other authors found a moderate [13] or even low association [14]. To make sense of the often conflicting results found, meta-analyses must be conducted [11,15,16]. Recently some meta-analytic studies have examined the criterion-related validity of different widely used physical fitness field tests such as the sit-and-reach [17], toe-touch [18], and 20-m shuttle run [7]. Regarding the distance- and time-based walk/run field tests, Safrit, Hooper, Ehlert, Costa, and Patterson [19] carried out the first meta-analysis almost 30 years ago. However, from that date many primary research studies have been published. Additionally, some walk/run tests were not taken into account. Since these authors performed the analysis combining all the tests, besides the well-known methodological problem of dependency, a key issue such as which test is “long enough” could not be addressed. Finally, apart from the sex and age of individuals, the potential moderator effects of other important issues on the criterion-related validity such as the individuals’ fitness levels, adding some other individuals’ characteristics to the performance score, or the measurement unit of the criterion test were not examined.

Unfortunately, to our knowledge there is not any recent meta-analysis examining the criterion-related validity of the distance- and time-based walk/run tests, and there is not any meta-analysis addressing all the above mentioned issues. Examining these questions would help to select the best feasible and valid test for estimating cardiorespiratory fitness. Consequently, the purposes of the present meta-analysis were: (a) to estimate and compare the overall population of the criterion-related validity of the distance- and time-based walk/run tests for estimating cardiorespiratory fitness among apparently healthy children and adults (for only performance score and performance score with other variables); (b) to examine the influence of individuals’ sex (men and women), age (children and adults), and level of VO_2_max (low and high) on criterion-related validity of the walk/run tests (between-study analyses); and (c) to compare the criterion-related validity between the only performance score and the performance score combined with other variables, as well as between the VO_2_max relative to body mass and the VO_2_max absolute, VO_2_max relative to fat-free mass and maximal aerobic speed (within-study analyses).

## Materials and Methods

The methodological procedure followed in the present study was based on previous general literature about meta-analyses [11,15,16], and specifically in the meta-analyses of the criterion-related validity of physical fitness field tests [7,17,18]. Although the present manuscript is original (including all the results, figures and tables) and data are from an independent study, it reproduces some parts of the text already published elsewhere [7,17,18].

### Data sources and search strategy

Seven electronic bibliographic databases were searched through until August 2015: Web of Science^™^ (all databases), Scopus, SPORTDiscus with Full Text, CINAHL, Cochrane Library, ProQuest Social Sciences Premium Collection, and ProQuest Dissertations & Theses Global. The searches were carried out in the search field type “Title, abstract, and keywords” or equivalent. The search terms used were based on two concepts: (1) walk/run field test, and (2) validity. The terms of the same concept were combined together with the Boolean operator “OR” and then the two concepts were combined using the Boolean operator ‘‘AND” [11]. The truncated root of certain terms was followed by an asterisk to include multiple variants. The keywords with more than one word were enclosed in quotes. Due to the large number of terms, from one to four independent searches were carried out for each walk/run field test. No publication format, language or date restrictions were imposed. See S1 Appendix for all the specific syntaxes used.

Based on the results of the Boolean-based database search, additional records were identified through other sources: (1) searching the reference lists of original studies and some related study reviews (i.e. “snowballing”); (2) examining the reference citations and the researchers publications (first authors) in the Web of Science^™^ and Scopus databases; (3) contacting by email with the corresponding authors (if they were not defined, the first author was used), and (4) screening the researchers’ personal lists in ResearchGate and Google Scholar (first authors). For practical reasons, the search was carried out for one researcher.

### Study selection

The selection criteria were the following: (1) studies with participants who did not present any injury, physical and/or mental disabilities; (2) studies with field tests performed on a track or similar (i.e. but not on a treadmill) that consisted of walking/jogging, walking/running, only jogging or only running (i.e. but not only walking) as much as possible during a fixed distance (i.e. 5,000 m, 3 miles, 2 miles, 3,000 m, 1.5 mile, 1 mile, 1,000 m, ½ mile, 600 m, 600 yd, and ¼ mile) or time (i.e. 15 min, 12 min, 9 min and 6 min); (3) studies in which for the criterion measure the VO_2_max (or VO_2_peak–see potentials and limitations section–) was measured in a standardized and laboratory-based graded exercise test to exhaustion, and (4) studies which reported (or could be computed from raw data reported in the study) the Pearson’s *r* zero-order correlation coefficient or simple/multiple linear regression (*R*^2^) of performance scores of the field test (or the performance score with other variables) with the measured VO_2_max.

Although there are many standard scales to assess the overall risk of bias in the included studies, empirical evidence has shown that they are misleading and unhelpful [20]. According to PRISMA and Cochraneʹs guidelines [21,22], the present meta-analysis followed a component approach on a case-by-case basis where it was described specific methodological domains that it was assessed. Both domains related to markers of the validity of the included studies (i.e. risk of bias in individual studies) and domains related to the research topic were included [21]. In addition to guaranteeing that the included studies met the selection criteria, in the present meta-analysis it was also ensured that there was a complete reporting of relevant outcomes. The sample size, protocol of the walk/run field test, unit and protocol of the criterion measure test, statistical test, and value of the criterion-related validity were considered to be critical. In the event that the authors failed to identify any critical study feature and it could not be retrieved, the study was not included in the meta-analysis. The selection criteria were examined by two independent researchers. When doubt or disagreement occurred (< 5%), a consensus was always achieved through discussion.

### Data extraction

From each selected study the following data were coded: Identification number, study reference, type of publication (1 = journal paper, 2 = grey literature–any document reporting some scientific finding, except the journal papers: e.g. doctoral dissertation, masterʹs thesis, conference proceeding, or technical report–), sample size (*n*), sex (1 = men, 2 = women, 3 = men and women), age (1 = children and adolescents–onwards the term “children” is used to simplify-, < 18 years, 2 = adults, ≥ 18 years, 3 = children and adults), field test (1 = 5,000 m, 2 = 3 miles, 3 = 2 miles, 4 = 3,000 m, 5 = 1.5 miles, 6 = 1 mile, 7 = 1,000 m, 8 = ½ mile, 9 = 600 m, 10 = 600 yd, 11 = ¼ mile, 12 = 15 min, 13 = 12 min, 14 = 9 min, 15 = 6 min), criterion measure protocol (1 = treadmill test, 2 = cycle ergometer test, 3 = other), measurement unit of the criterion test (1 = VO_2_max relative to body mass, 2 = VO_2_max absolute, 3 = VO_2_max relative to fat-free mass, 4 = maximal aerobic speed, 5 = other), mean and standard deviation values of the measurement criterion, reliability of the field test and the measurement criterion (intraclass correlation coefficient), statistical test (1 = Pearson’s *r* correlation coefficient, 2 = *R*^2^ simple/multiple linear regression), and criterion-related validity value (separately for only performance score and multiple predictors). Observations were also registered when special issues were found. In the event that the authors failed to identify any study feature, they were contacted to retrieve it. If the study feature was not retrieved, the information was omitted. Coding studies were carried out by two independent researchers. When doubt or disagreement occurred (< 5%), a consensus was always achieved through discussion.

### Data analyses

A detailed description of the data analyses carried out in the present meta-analysis [16], as well as a brief description of the main formulas [11], can be found elsewhere. According to Schmidt and Hunter [16], Pearson’s zero-order correlation coefficient (*r*) was considered the unit of the criterion-related validity of the walk/run field tests. When the validity values were reported as *R*^2^, it was previously transformed by the square root. After verifying that in all the primary studies a better performance in the walk/run field tests (i.e. more distance in the time-based tests, less time in the distance-based tests or higher average speed in both tests) was associated with a better score in the criterion measure, the correlation coefficients between the time score of the distance-based walk/run tests and the criterion measure (i.e. negative values) were previously transformed to positive (i.e. absolute values). The studies carried out with a small sample (defined as less than 10 participants) were not included.

#### Dependency issues

An exhaustive examination of the selected studies was carried out to avoid dependency issues. Since the most studies used the VO_2_max relative to body mass as the measurement criterion, the correlation coefficients with this variable were used for the main analyses. When these studies also reported the results of criterion-related validity using additional variables (i.e. the VO_2_max absolute, VO_2_max relative to fat-free mass and/or the maximal aerobic speed), these validity coefficients were only used for the within-study analyses to compare with the VO_2_max relative to body mass. Since some studies used multiple performance scores of the field tests for examining the criterion-related validity, the average value was used. When authors reported the results of criterion-related validity from the combination of different multiple predictors, only the best model (i.e. higher coefficient value) was used.

If a single study reported more than one *r* value within the same field test, but from different subsamples, each *r* value from different subsamples was assumed to be independent [15]. When, in the same study, data for men/women or children/adults were expressed both separately and together, only the separate data were selected. However, when data for the whole sample and subsamples with respect to sex and age categories were expressed, only the whole sample was used. When data were expressed for different trials, the average value of the coefficients was selected. When data were expressed for pre- and post-intervention, only the pre-intervention value was used.

#### Publication bias

Besides the search strategy followed to avoid availability bias, a deep examination of the selected studies was first carried out to avoid any potential duplication of the information retrieved. Similarities between studies of the same authors, with the same correlation coefficients and/or the same sample size were examined. When the selected studies had full or partial duplicated information, these particular correlations were not analyzed. Then, to identify the impact of any potential publication bias, the scatter plots and the Spearman’s rank order correlations between *r* values and sample size were carried out [23]. Cumulative meta-analyses by year of publication were also examined through forest plots to assess the evolution of the summary of the criterion-related validity coefficients over time [23]. Finally, for assessing the impact of any potential publication bias, file drawer analyses based on effect size were performed [23]. According to Cohen’s [24] benchmarks, in the file drawer analyses a small correlation coefficient was defined as *r* = 0.29.

#### Computation of correlations

The Hunter-Schmidt’s psychometric meta-analysis approach [16] was conducted to obtain the population estimates of the criterion-related validity of the walk/run field tests. These authors advocate a single method (a random-effects model) on the basis of their belief that a fixed-effects model is inappropriate for real world data and the type of inferences that researchers usually want to make [16]. Schmidt and Hunter [16] also argue that when the random-effects model is applied to data in which the same *p* value (i.e. population parameter of *r*) underlies all studies (i.e. SD_p_ = 0), it becomes mathematically a fixed-effects model. That is, while the random-effects model is a more general method that allows for any possible value of SD_p_, the fixed-effects model allows for only one special case, i.e. when SD_p_ = 0.

The “bare-bone” mean *r* (*r*_c_), corrected for only sampling error was first calculated by weighting each *r* with the respective sample size. Then, the corrected mean *r* at the population level (*r*_p_) that was unaffected by both sampling error and measurement error was calculated. Since the reliability coefficients of the field tests were unavailable in most of the included primary studies, the measurement error was corrected using artifact distributions. The measurement error of the criterion test could not be corrected because the reliability was almost unavailable. Finally, the 95% confidence intervals of *r*_p_ (95% CI) were calculated.

#### Moderator analyses

According to Schmidt and Hunter [16], to determine the presence of heterogeneity in the population estimation of the criterion-related validity of the field tests (*r*_p_), three different criteria were simultaneously examined: (a) the 95% credibility interval (95% CV) is relatively large or includes the value zero; (b) the percentage of variance accounted for by statistical artefacts is less than 75% of the observed variance in *r*_p_, and (c) the *Q* homogeneity statistic is statistically significant at *p* < 0.05. If at least one of the three criteria was met, it was concluded that the results were potentially affected by moderator effects.

Based on *a priori* hypothesized moderators, partial hierarchical analyses were also carried out (i.e. subgroups or stratified analyses). The criterion-related validity of the walk/run field tests were analyzed by: (a) sex (i.e. men and women); (b) age (i.e. children and adults); and (c) level of VO_2_max (i.e. low average level, < P_50_, and high average level, ≥ P_50_) (between-study analyses). Additionally, the criterion-related validity of the field tests for the only performance score and multiple predictors was compared; and the criterion-related validity with the VO_2_max relative to body mass were compared to the VO_2_max absolute, VO_2_max relative to fat-free mass, and maximal aerobic speed (within-study analyses).

The meta-analyses were performed using the software Hunter and Schmidt Meta-Analysis Programs version 2.0 for Windows (Iowa, 2014). All the others statistical analyses and graphs were performed using the SPSS version 20.0 for Windows (IBM^®^ SPSS^®^ Statistics 20).

## Results

### Study description

**Fig 1:**
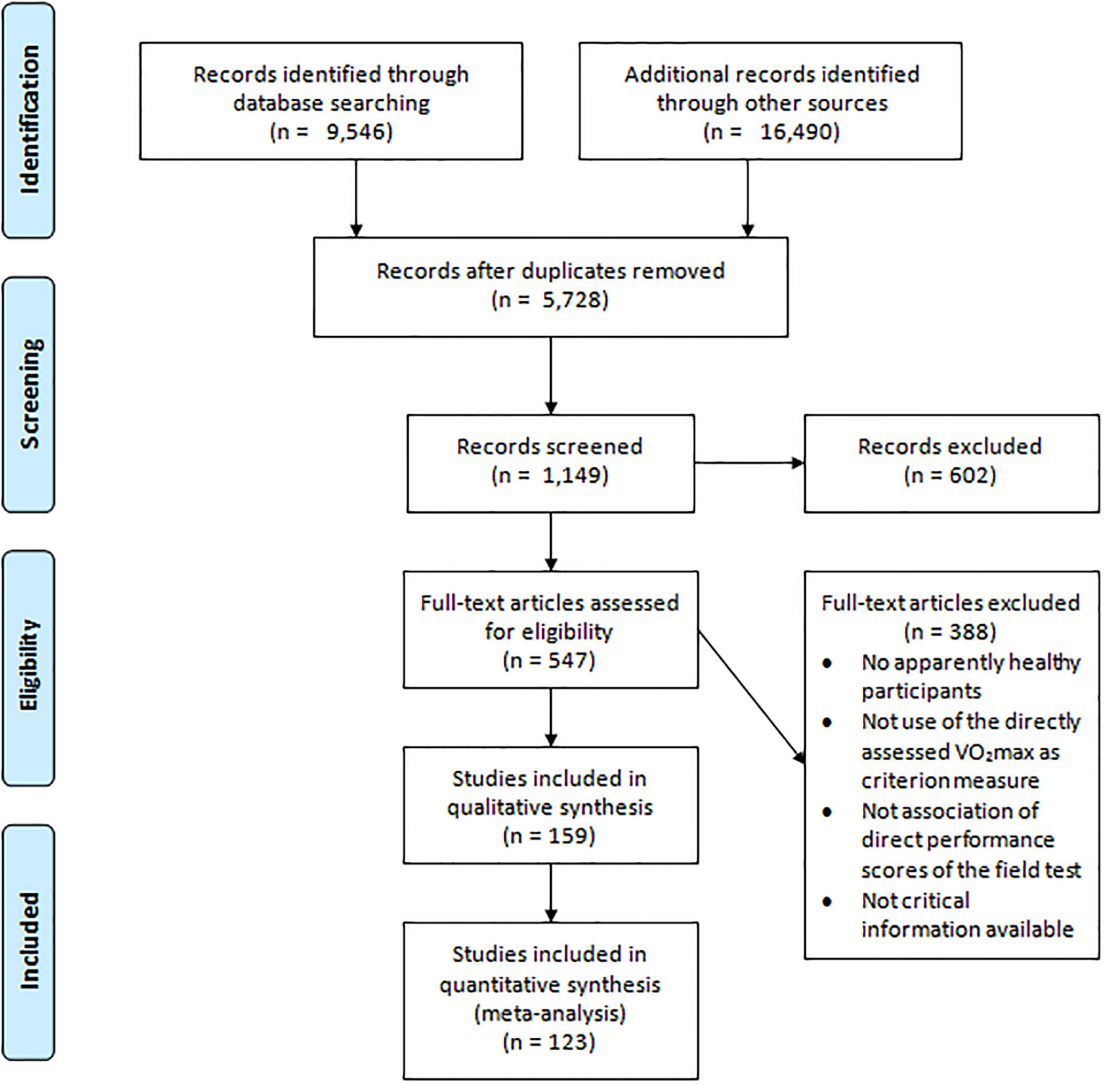
Flow diagram of the study selection process.

Of the 9,546 bibliographic databases search results, potentially relevant publications were retrieved for a more detailed evaluation. Afterward, based on the studies of the Boolean-based database search, additional records were identified through other sources. From the 547 potentially eligible studies, 159 studies met the selection criteria. However, due to full duplication issues, not reporting the criterion-related validity of the VO_2_max relative to body mass and/or carrying out the study with a small sample, only 123 studies were included [10,12–14,25–143]. From the included studies, 200 *r* values across the walk/run field tests were retrieved, being 178 correlation coefficients for the criterion-related validity using the only performance score and 22 for multiple predictors (Fig 1).

Regarding the criterion-related validity for only performance score, a total of 178 *r* values across 15 walk/run tests were retrieved, ranging from 1 to 34 values (median = 9). Total sample sizes for each field test ranged from 28 to 1,856 (median = 367). The individual criterion-related validity ranged from 0.03 to 0.99 (median = 0.70). Regarding the criterion-related validity for performance with other variables, a total of 22 *r* values across eight walk/run tests were retrieved, ranging from 1 to 6 values (median = 1). Total sample sizes for each field test ranged from 44 to 1,156 (median = 87). The individual criterion-related validity ranged from 0.65 to 0.99 (median = 0.81) (S1 Table).

### Publication bias

#### Avoiding duplicated information

Although 16 research studies met the selection criteria, the correlation coefficients were not analyzed. Some grey literature sources were not included because they were published later in a journal paper, e.g. [144–146]. From the Cureton’s et al. [51] study the correlation coefficient with the only performance score was not included because the data came from the sum of some samples that had been reported in other journal papers [49,50,96]. However, since these papers did not report the correlation coefficient with multiple predictors, the correlation coefficient with multiple predictors for the overall results and both the only performance score and multiple predictors for the within-study analysis from the Cureton’s et al. [51] study were used.

#### Identifying publication bias

**Fig 2:**
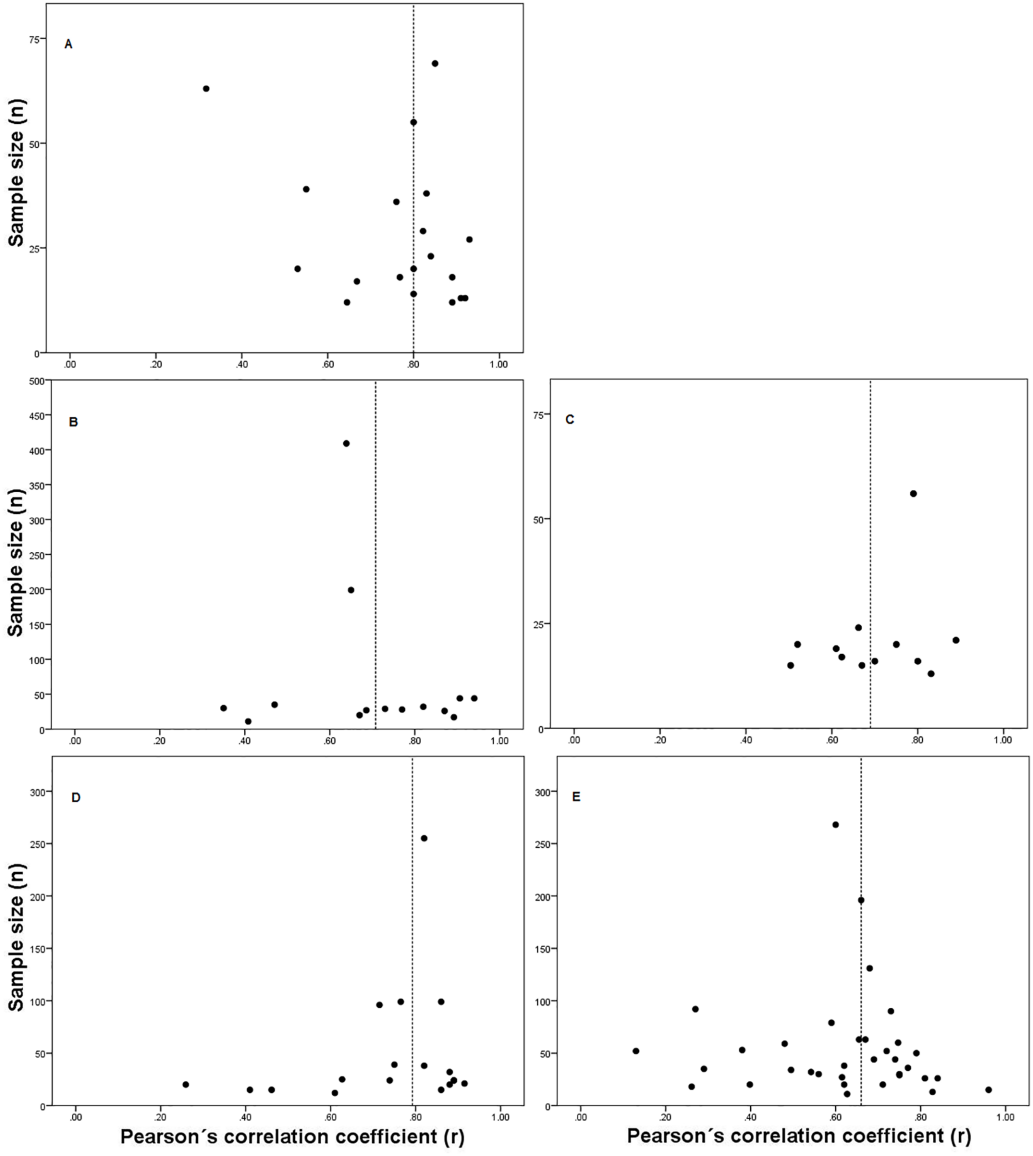
Scatter plot of sample size against criterion-related validity coefficients (*r*) of distance-based walk/run tests for estimating maximal oxygen uptake: (a) 5,000 m walk/run test; (b) 2 miles walk/run test; (c) 3,000 m walk/run test; (d) 1.5 mile walk/run test; and (e) 1 mile walk/run test. Dashed line represents median values of validity coefficients.

**Fig 3:**
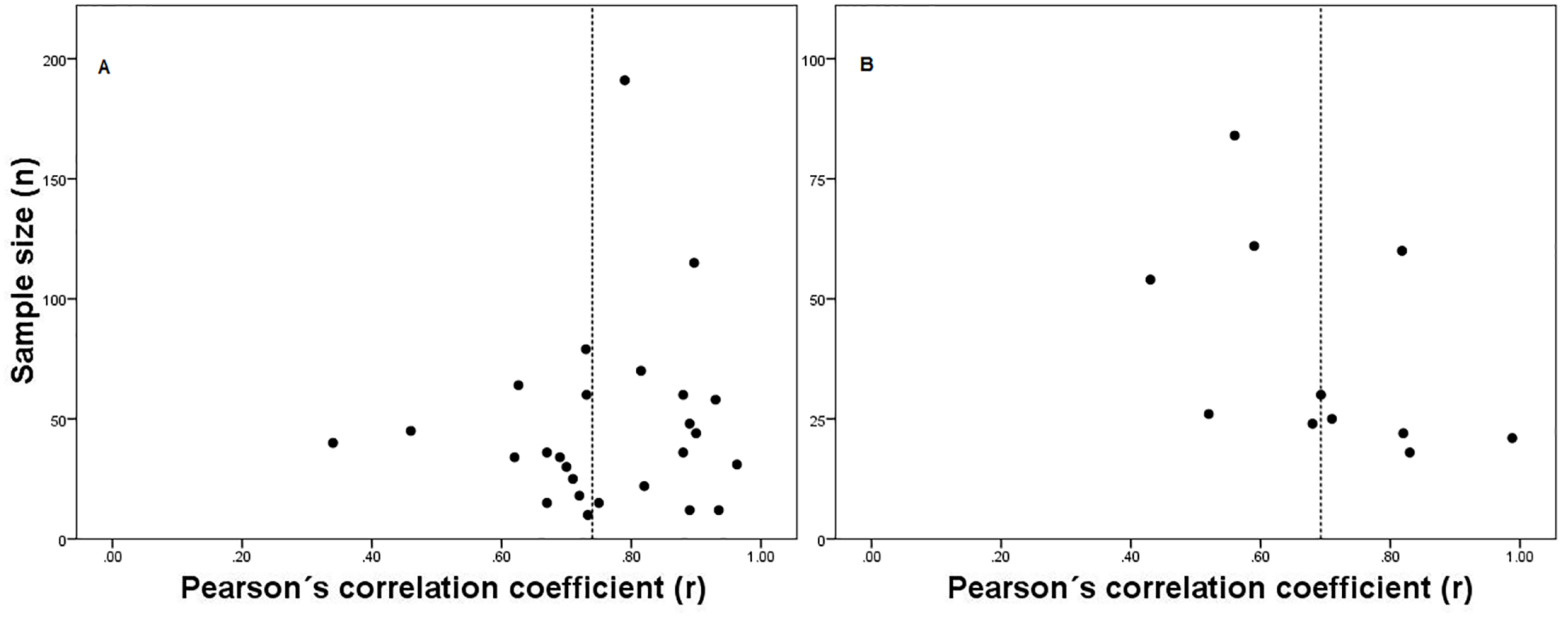
Scatter plot of sample size against criterion-related validity coefficients (*r*) of time-based walk/run tests for estimating maximal oxygen uptake: (a) 12 min walk/run test; and (b) 9 min walk/run test. Dashed line represents median values of validity coefficients.

The following analyses were calculated only for the tests with a *K* equal to 10 or more [147]. The scatter plots of sample size against criterion-related validity coefficients suggest that for the distance-based walk/run tests there was not publication bias (Fig 2). For the time-based walk/run tests explored, however, the figures suggest the presence of publication bias because of the absence of *r* values in the lower left hand corner (Fig 3). Similarly, while the results of Spearman’s rank order correlation between *r* values and sample size did not show any statistically significant correlation for the distance-based walk/run tests (*p* > 0.05), a statistically significant correlation was found for the 9 min walk/run test (*p* < 0.05). However, for the 12 min walk/run test a statistically significant correlation was not found (*p* > 0.05). Due to the small *K* found for most of the tests, the results of both methods must be interpreted with caution [11,23]. Empirical evaluations of the funnel plots also suggest that their interpretation can be limited [148].

In the walk/run tests analyzed (i.e. *K* ≥ 10), the cumulative meta-analysis plots (S1 Fig) suggest a fairly constant estimate of the criterion-related validity coefficients over time with only some fluctuations in the first studies may be simply due to chance [23]. Although a large correlation coefficient in the first primary study was found for the 12 min walk/run test (S1F Fig), the summary value was diminished after the 3rd-4th study. Additionally, no transiently lose formal significance nor complete reverse of the initial association was found. Finally, it is worth mentioning that in most plots (i.e. S1C–S1F Fig) the addition of new primary studies did not materially change the estimates, consequently the final criterion-related validity values of these walk/run tests seem to be quite robust. On the other hand, for the other walk/run tests (i.e. S1A, S1B and S1G Fig and *K* < 10) the final criterion-related validity values should be considered with special caution.

#### Assessing the impact of publication bias

The results of the file drawer analyses are shown in the following lines (in parenthesis the unlocated/located percentage): 5,000 m 28 (147%), 3 miles 6 (100%), 2 miles 19 (136%), 3,000 m 16 (133%), 1.5 miles 30 (167%), 1 mile 36 (106%), 1,000 m 3 (150%), 600 m 1 (100%), 600 yd 8 (100%), ¼ mile 5 (63%), 15 min 1 (50%), 12 min 42 (162%), 9 min 14 (127%), and 6 min 7 (88%). The results showed an unlikely number of “lost” studies for most of the walk/run tests, especially if the percentage of unlocated/located studies is considered (73–167%).

### Criterion-related validity

The overall results showed that the criterion-related validity of the walk/run tests with the only performance score ranged from low to moderate and no 95% CI included the value zero. The results also showed that the criterion-related validity of the 1.5 mile and 12 min walk/run tests was statistically significantly higher than the 3 miles, 1 mile, ½ mile, 600 yd, ¼ mile, 15 min, and 6 min walk/run tests (*p* < 0.05). The 5,000 m walk/run test was statistically significantly greater than the ¼ mile, 15 min and 6 min walk/run tests (*p* < 0.05). And the 2 miles and 3,000 m walk/run tests showed a statistically significant higher mean *r* than the 6 min walk/run test (*p* < 0.05). For the other comparisons statistically significant differences were not found (*p* > 0.05) (Table 1). Since for most of the tests at least one heterogeneity criterion was met (Table 1), follow-up moderator analyses were conducted. Due to the small *K*, moderator analyses were not conducted for the 1,000 m, 600 m and 15 min walk/run tests.

**Table 1:** Results of meta-analyses for overall criterion-related validity correlation coefficients across the distance- and time-based walk/run field tests.

Protocols	*K*	*N*	*r*_c_	*r*_p_	95% CI^a^	95% CV^b^	% variance^c^	*Q* statistic
*Only performance score*								
5,000 m	19	536	0.70	0.73	0.65–0.81	0.44–1.00	31.11	43.96*
3 miles	6	177	0.58	0.60	0.48–0.72	0.45–0.75	73.18	2.30
2 miles	14	951	0.66	0.69	0.62–0.75	0.50–0.88	34.72	27.51*
3,000 m	12	252	0.67	0.70	0.63–0.76	0.70–0.70	100.00	0.00
1.5 mile	18	873	0.76	0.79	0.73–0.85	0.57–1.00	24.74	57.21*
1 mile	34	1,856	0.60	0.62	0.56–0.67	0.36–0.88	32.05	75.32*
1,000 m	2	71	0.72	0.74	0.46–1.00	0.38–1.00	17.25	10.02*
½ mile	9	241	0.53	0.55	0.45–0.65	0.46–0.63	91.49	0.88
600 m	1	28	0.54	0.56	-	-	-	-
600 yd	8	415	0.58	0.60	0.50–0.70	0.39–0.81	44.65	10.36
¼ mile	8	232	0.44	0.45	0.26–0.64	0.02–0.89	33.22	16.80*
15 min	2	120	0.41	0.42	0.21–0.64	0.22–0.62	54.24	1.76
12 min	26	1,204	0.75	0.78	0.72–0.83	0.54–1.00	22.56	93.24*
9 min	11	425	0.63	0.66	0.57–0.74	0.46–0.86	49.16	11.89
6 min	8	367	0.54	0.55	0.49–0.62	0.55–0.55	100.00	0.00
*Performance score with other variables*								
2 miles	1	44	0.94	0.97	-	-	-	-
1.5 mile	4	210	0.87	0.90	0.86–0.95	0.84–0.96	55.72	3.32
1 mile	6	1,156	0.76	0.79	0.74–0.84	0.68–0.89	26.13	17.73*
½ mile	1	47	0.66	0.69	-	-	-	-
600 yd	1	53	0.65	0.68	-	-	-	-
12 min	3	169	0.78	0.81	0.73–0.89	0.72–0.90	56.65	2.40
9 min	5	283	0.76	0.79	0.74–0.84	0.79–0.79	100.00	0.00
6 min	1	87	0.77	0.80	-	-	-	-

*Note*. *K*, number of *r*s; *N*, total sample size; *r*_c_, overall weighted mean of *r* corrected for sampling error only; *r*_p_, overall weighted mean of *r* corrected for sampling error and measurement error of the field tests;

^a^95% confidence interval;

^b^95% credibility interval;

^c^Percentage of variance accounted for by statistical artefacts including sampling error and measurement error of the field tests.

* *p* < 0.05

Regarding the multiple predictors, the overall results showed that when the performance score of the walk/run field tests was combined with other variables the criterion-related validity values were moderate-to-very-high and no 95% CI included the value zero. The results also showed that the criterion-related validity of the 1.5 mile walk/run test was statistically significantly higher than the 1 mile and 9 min walk/run tests (*p* < 0.05). However, statistically significant differences between the 1.5 mile and 12 min walk/run tests were not found (*p* > 0.05), as well as neither between the 1 mile, 12 min, and 9 min walk/run tests (*p* > 0.05) (Table 1). Although at least one heterogeneity criterion was met in three of the four tests (Table 1), due to the small *K* the between-study moderator analyses were not conducted for multiple predictors. The within-study analyses were conducted as it was hypothesized (see moderator analyses).

### Moderator analyses

#### Between-study moderator analyses

The results of the between-study moderator analyses showed that the criterion-related validity of the analyzed walk/run field tests ranged from low to moderate for all the subcategories. No 95% CI included the value zero, except for the 600 yd walk/run test in women and the ¼ mile walk/run test in individuals with high level of VO_2_max. Regarding the within-test comparisons between men-women, children-adults and low-high level of VO_2_max, statistically significant differences were not found (*p* > 0.05) (except for the 9 min walk/run test in the level of VO_2_max category). As regards the between-test comparisons among each subcategory, in general the results showed that the criterion-related validity of the 1.5 mile and 12 min walk/run tests was statistically significantly higher than those tests with shorter protocols (*p* < 0.05). Nevertheless, no statistically significant differences were found between the criterion-related validity of the 1.5 mile and 12 min walk/run tests and the walk/run tests with longer protocols (except among the adults in which for the 1.5 mile walk/run test was statistically significantly higher than the 3 mile walk/run tests, *p* < 0.05). According to heterogeneity analyses, at least one criterion was met in most of the walk/run tests, indicating that the criterion-related validity of these tests separately for sex, age and level of VO_2_max was still heterogeneous. Because some studies grouped subcategories together or values were missing, overall *K* for the categories is lower (Table 2).

**Table 2:** Results of the between-study moderator analyses for criterion-related validity correlation coefficients across the distance- and time-based walk/run field tests†.

Moderator	Effect	*K*	*N*	*r*_c_	*r*_p_	95% CI^a^	95% CV^b^	% variance^c^	*Q* statistic
*Sex of participants*									
5,000 m	Men	10	302	0.67	0.69	0.56–0.82	0.34–1.00	26.49	28.99*
	Women	5	139	0.80	0.83	0.80–0.85	0.83–0.83	100.00	0.00
3 miles	Men	5	162	0.58	0.60	0.46–0.73	0.41–0.78	61.64	3.25
	Women	1	15	0.66	0.68	-	-	-	-
2 miles	Men	9	816	0.64	0.66	0.59–0.73	0.50–0.82	39.22	14.58
	Women	4	109	0.81	0.84	0.73–0.95	0.67–1.00	39.57	6.38
3,000 m	Men	9	196	0.67	0.69	0.63–0.76	0.69–0.69	100.00	0.00
	Women	3	56	0.70	0.72	0.55–0.89	0.55–0.89	67.71	1.50
1.5 mile	Men	12	585	0.75	0.78	0.70–0.86	0.54–1.00	22.22	43.90*
	Women	4	150	0.74	0.77	0.66–0.88	0.60–0.94	44.33	5.25
1 mile	Men	15	591	0.58	0.60	0.50–0.69	0.31–0.88	36.52	27.24*
	Women	10	415	0.55	0.57	0.43–0.70	0.20–0.93	26.60	28.83*
½ mile	Men	5	125	0.55	0.57	0.42–0.71	0.44–0.69	84.51	0.96
	Women	2	30	0.45	0.46	0.09–0.84	0.16–0.76	66.98	1.03
600 yd	Men	4	158	0.60	0.62	0.50–0.73	0.53–0.71	83.70	0.81
	Women	2	46	0.30	0.31	-0.01–0.63	0.09–0.54	75.30	0.69
¼ mile	Men	3	60	0.24	0.25	0.10–0.40	0.25–0.25	100.00	0.00
	Women	-	-	-	-	-	-	-	-
12 min	Men	13	761	0.76	0.79	0.71–0.87	0.51–1.00	13.75	85.19*
	Women	8	285	0.75	0.78	0.70–0.85	0.64–0.91	54.47	6.99
9 min	Men	6	176	0.69	0.72	0.62–0.81	0.59–0.85	69.83	2.71
	Women	4	165	0.61	0.63	0.46–0.80	0.36–0.90	34.87	7.81*
6 min	Men	3	126	0.53	0.54	0.44–0.64	0.54–0.54	100.00	0.00
	Women	2	55	0.49	0.50	0.39–0.62	0.50–0.50	100.00	0.00
*Age of participants*									
5,000 m	Children	1	12	0.65	0.67	-	-	-	-
	Adults	18	524	0.71	0.73	0.65–0.81	0.43–1.00	29.27	45.44*
3 miles	Children	-	-	-	-	-	-	-	-
	Adults	5	150	0.56	0.58	0.44–0.72	0.41–0.75	70.26	2.21
2 miles	Children	-	-	-	-	-	-	-	-
	Adults	13	924	0.67	0.69	0.62–0.75	0.49–0.88	32.21	28.58*
3,000 m	Children	2	32	0.65	0.67	0.54–0.80	0.67–0.67	100.00	0.00
	Adults	10	220	0.68	0.70	0.63–0.77	0.70–0.70	100.00	0.00
1.5 mile	Children	3	234	0.73	0.76	0.73–0.78	0.76–0.76	100.00	0.00
	Adults	15	639	0.77	0.80	0.72–0.87	0.54–1.00	19.71	63.85*
1 mile	Children	22	1,499	0.58	0.60	0.53–0.66	0.34–0.85	28.99	56.31*
	Adults	11	330	0.68	0.71	0.61–0.80	0.47–0.94	41.74	16.05
½ mile	Children	5	143	0.47	0.48	0.37–0.60	0.48–0.48	100.00	0.00
	Adults	4	98	0.62	0.64	0.51–0.77	0.61–0.67	98.66	0.06
600 yd	Children	7	371	0.56	0.57	0.47–0.68	0.39–0.76	52.91	6.51
	Adults	1	44	0.78	0.81	-	-	-	-
¼ mile	Children	2	86	0.55	0.57	0.45–0.69	0.57–0.57	100.00	0.00
	Adults	6	146	0.37	0.38	0.13–0.64	-0.13–0.89	33.36	12.52*
12 min	Children	8	246	0.69	0.71	0.67–0.76	0.71–0.71	100.00	0.00
	Adults	17	767	0.76	0.79	0.71–0.87	0.49–1.00	14.99	100.78*
9 min	Children	10	407	0.63	0.65	0.56–0.74	0.45–0.85	48.19	11.24
	Adults	1	18	0.83	0.85	-	-	-	-
6 min	Children	7	337	0.52	0.53	0.48–0.59	0.53–0.53	100.00	0.00
	Adults	1	30	0.79	0.81	-	-	-	-
*Level of VO*_*2*_*max*									
5,000 m	Low	9	241	0.74	0.76	0.69–0.83	0.68–0.85	82.41	2.01
	High	10	295	0.68	0.70	0.56–0.84	0.32–1.00	22.76	35.46*
3 miles	Low	2	50	0.64	0.66	0.34–0.98	0.29–1.00	30.32	4.80*
	High	3	92	0.61	0.63	0.57–0.68	0.63–0.63	100.00	0.00
2 miles	Low	6	562	0.69	0.71	0.63–0.80	0.55–0.88	30.46	14.31*
	High	7	354	0.65	0.67	0.57–0.76	0.48–0.85	45.82	8.65
3,000 m	Low	6	115	0.67	0.69	0.60–0.79	0.69–0.69	100.00	0.00
	High	6	137	0.68	0.70	0.61–0.79	0.70–0.70	100.00	0.00
1.5 mile	Low	8	556	0.79	0.81	0.72–0.90	0.58–1.00	13.93	51.66*
	High	9	293	0.71	0.74	0.66–0.81	0.62–0.85	69.71	4.09
1 mile	Low	16	899	0.63	0.65	0.57–0.73	0.37–0.93	25.67	48.41*
	High	16	902	0.58	0.60	0.53–0.67	0.40–0.80	44.16	21.14
½ mile	Low	4	70	0.54	0.55	0.36–0.75	0.38–0.73	80.63	1.00
	High	5	171	0.53	0.54	0.43–0.66	0.54–0.54	100.00	0.00
600 yd	Low	4	257	0.56	0.58	0.43–0.74	0.33–0.84	31.75	8.99*
	High	4	158	0.60	0.62	0.50–0.73	0.53–0.71	83.70	0.81
¼ mile	Low	3	97	0.50	0.51	0.31–0.71	0.31–0.72	62.95	1.85
	High	3	69	0.28	0.29	-0.02–0.60	-0.06–0.64	56.13	2.45
12 min	Low	8	246	0.69	0.71	0.67–0.76	0.71–0.71	100.00	0.00
	High	12	629	0.73	0.75	0.66–0.84	0.48–1.00	19.18	52.83*
9 min	Low	5	213	0.54	0.56	0.48–0.64	0.56–0.56	100.00	0.00
	High	6	212	0.73	0.75	0.66–0.85	0.59–0.92	48.15	6.75
6 min	Low	4	227	0.54	0.55	0.46–0.65	0.51–0.60	95.24	0.21
	High	4	140	0.54	0.55	0.47–0.64	0.55–0.55	100.00	0.00

*Note*. *K*, number of *r*s; *N*, total sample size; *r*_c_, overall weighted mean of *r* corrected for sampling error only; *r*_p_, overall weighted mean of *r* corrected for sampling error and measurement error of the field tests; VO_2_max, maximal oxygen uptake;

^a^95% confidence interval;

^b^95% credibility interval;

^c^Percentage of variance accounted for by statistical artefacts including sampling error and measurement error of the field tests.

^†^Because some studies mixed categories or data were missing, the overall *K* for some categories is lower for some field tests.

* *p* < 0.05

#### Within-study moderator analyses

Because of the low *K* for each field test, the within-study analyses were carried out with all the tests together. As regards the analyses for the number of predictors, the results showed that meanwhile the only performance score had a moderate criterion-related validity, when other variables were added the criterion-related validity values were moderate-to-high. No 95% CI included the value zero. The criterion-related validity of the performance score with other variables (i.e. multiple predictors) was statistically significantly higher than only the performance score (*r*_p_Δ = 0.14; *p* < 0.05) (Table 3).

**Table 3:** Results of the within-study moderator analyses for criterion-related validity correlation coefficients across the distance- and time-based walk/run field tests†.

Effect	*K*	*N*	*r*_c_	*r*_p_	95% CI^a^	95% CV^b^	% variance^c^	*Q* statistic
*Number of predictors*^d^								
One predictor	16	1,400	0.63	0.65	0.59–0.71	0.46–0.84	32.45	34.80*
Multiple predictors	16	1,400	0.76	0.79	0.75–0.82	0.67–0.90	37.44	27.93*
*VO*_*2*_*max absolute*								
VO_2_max (ml/kg/min)	32	1,809	0.61	0.63	0.59–0.68	0.46–0.81	47.78	36.55
VO_2_max (l/min)	32	1,809	0.36	0.37	0.32–0.42	0.25–0.49	80.59	8.05
*VO*_*2*_*max relative to fat-free mass*								
VO_2_max (ml/kg/min)	16	864	0.67	0.70	0.65–0.74	0.63–0.76	85.45	2.85
VO_2_max (ml/kg FFM/min)	16	864	0.48	0.50	0.42–0.58	0.27–0.73	46.49	19.24
*Maximal aerobic speed*								
VO_2_max (ml/kg/min)	11	176	0.57	0.59	0.47–0.70	0.43–0.74	84.13	2.17
MAS (km/h)	11	176	0.68	0.71	0.60–0.81	0.51–0.90	67.30	5.59

*Note*. *K*, number of *r*s; *N*, total sample size; *r*_c_, overall weighted mean of *r* corrected for sampling error only; *r*_p_, overall weighted mean of *r* corrected for sampling error and measurement error of the field tests;

^a^95% confidence interval;

^b^95% credibility interval;

^c^Percentage of variance accounted for by statistical artefacts including sampling error and measurement error of the field tests;

^d^Only performance score (“one predictor”) or performance score plus other variables (“multiple predictors”); VO_2_max, maximal oxygen uptake; FFM, fat-free mass; MAS, maximal aerobic speed.

^†^Because of the low *K* in each field test, the overall results are reported.

* *p* < 0.05

Regarding the analyses for the unit of the criterion measure, the results showed that the criterion-related validity values of the walk/run tests with the VO_2_max relative to body mass and maximal aerobic speed were moderate, but when the VO_2_max absolute and relative to fat-free mass was used instead it was low. No 95% CI included the value zero. The criterion-related validity of the walk/run tests with the VO_2_max relative to body mass was statistically significantly higher than when the VO_2_max absolute (*r*_p_Δ = 0.27; *p* < 0.05) or relative to fat-free mass was used (*r*_p_Δ = 0.20; *p* < 0.05). However, statistically significant differences between the VO_2_max relative to body mass and maximal aerobic speed were not found (*r*_p_Δ = - 0.12; *p* > 0.05). According to heterogeneity analyses, at least one criterion was met in each subcategory, indicating that the criterion-related validity of the walk/run tests was heterogeneous (Table 3). The fact that the different walk/run tests were put together must be also taken into account.

## Discussion

A cardiorespiratory fitness test must be chosen based on its feasibility and validity [7]. Although many distance- and time-based walk/run field tests have been proposed [8], according to the results of the present meta-analysis, the 1.5 mile and 12 min walk/run tests showed the greater criterion-related validity for estimating the cardiorespiratory fitness. The overall criterion-related validity of both tests has shown to be similar to other cardiorespiratory fitness tests such as the 20-m shuttle run test (*r*_p_ = 0.84, 0.80–0.89) [7].

According to the findings of the present meta-analysis, sex, age, and fitness levels of individuals do not seem to affect the criterion-related validity. Therefore, the walk/run tests can be used interchangeably for any subcategory. Similarly, recently Mayorga-Vega et al. [7], carrying out a meta-analytic study about the criterion-related validity of the 20-m shuttle run test, found that sex and fitness levels did not affect the validity. However, they found out that the criterion-related validity of the Léger’s protocol was statistically significantly higher among adults than among children. Although among children the 1.5 mile and 12 min walk/run tests showed a similar validity than the 20-m shuttle run test (*r*_p_ = 0.78, 0.72–0.85), among adults the 20-m shuttle run test was statistically significantly higher (*r*_p_ = 0.94, 0.87–1.00). Therefore, among adults the 20-m shuttle run test should be used instead the walk/run field tests.

A potential reason for these differences could be inherent to the protocols of the field tests. Meanwhile in the walk/run tests individuals have to run as much as possible maintaining a self-pace, the 20-m shuttle run test is characterized to have a rigid standardized protocol where individuals cannot choose their own pace. Specifically, it has been suggested that the starting speed of the 20-m shuttle run test could be too high for children [149]. Current evidence suggests that to elicit valid VO_2_max values, continuous incremental tests should last at least five minutes [150]. However, Castro-Piñero et al. [151] in a population-based study carried out with the Léger’s protocol (i.e. starting speed at 8.5 km/h) found that most children lasted less than five minutes. Thus, meanwhile with the walk/run tests both children and adults can adjust the running pace to their own possibilities, the most widely used protocols of the 20-m shuttle run test [152–154] could be too high for children. In this line, recent studies have proposed modifications of the 20-m shuttle run test for children with a drastically reduced starting speed (e.g. 4 km/h or 6.5 km/h) [107,149]. Future studies should compare the criterion-related validity of 1.5 mile and/or 12 min walk/run field tests and a modified version of the 20-m shuttle run test with a lower starting speed among children.

For both men-women, children-adults and low-high level of VO_2_max subgroups, the 1.5 mile and 12 min walk/run tests seem to be the most appropriate distance- and time-based walk/run field tests, respectively. Although longer distance-based field tests showed similar criterion-related validity results, performing a longer distance seems to be an unnecessary extra time and effort. However, due to their lower criterion-related validity, the use of shorter walk/run tests should be avoided. Surprisingly, among children the 1 mile walk/run test (followed by the ½ mile and ¼ mile walk/run tests) is the cardiorespiratory fitness test more often proposed by the field-based physical fitness batteries [8]. For instance, the FITNESSGRAM^®^ test battery proposes the use of either the 20-m shuttle run or 1 mile walk/run tests [9]. According to the results of the present meta-analysis, however, in addition to the 20-m shuttle run test, the 1.5 mile and/or 12 min walk/run tests should be proposed instead of the 1 mile walk/run test for estimating cardiorespiratory fitness among children.

The results of the present meta-analysis also showed that when multiple predictors were used, the criterion-related validity was statistically significantly higher than for the only performance score. Therefore, apart from the running performance score, adding other individuals’ variables significantly improves the estimation of the VO_2_max. Similarly, Mayorga-Vega et al. [7] found that for the 20-m shuttle run test with multiple predictors the correlation coefficient was considerably higher than for the only performance score (*r*_p_Δ = 0.11). However, probably because of the low number of correlations, this difference was not statistically significant. Another potential reason for these differences could be due to the fact that the validity of the walk/run tests was lower than the 20-m shuttle run test and, therefore, the change to increase the explained variance was greater.

Finally, the results of the present meta-analysis showed that the criterion-related validity of the walk/run tests with the VO_2_max relative to body mass as the measurement unit was statistically significantly higher than when the VO_2_max absolute or relative to fat-free mass was used. According to Meredith and Welk [9], the criterion-related validity of walk/run tests with VO_2_max relative to body mass should not be interpreted only in terms of cardiorespiratory fitness, but they also reflect the influence of differences on body fat. In this line, empirical evidence has demonstrated that part of the association of VO_2_max relative to body mass with the walk/run tests reflects the influence of anthropometric variables [155]. Therefore, it is not surprising the fact that the correlation of the walk/run tests with the VO_2_max expressed relative to body mass is higher than with the VO_2_max expressed absolute or relative to fat-free mass. On the other hand, statistically significant differences between the VO_2_max relative to body mass and maximal aerobic speed were not found. The maximal aerobic speed defined as the lowest speed at which VO_2_max occurs, besides the differences in body mass previously mentioned, it reflects other factors such as running economy. Although running economy influences the running performance in a walk/run test, it has shown not to increase the variance explained between the walk/run test score and the VO_2_max relative to body mass [50].

### Potentials and limitations

The meta-analysis is a useful tool to assess scientific evidence, but an understanding of its potentials and limitations is needed. An exhaustive review of the general potentials and limitations of meta-analyses, e.g. [11], as well as specifically in the meta-analysis of the criterion-related validity of cardiorespiratory fitness field tests has been published elsewhere [7]. Regarding the potentials of the present meta-analysis, numerous measures to avoid, or at least to reduce, publication bias were followed. Then, several exploratory analyses were conducted to identify and assess the impact of any potential publication bias. Another potential was related to the statistical approach used. Since the Hunter-Schmidt’s psychometric meta-analysis approach [16] estimates the population correlation by correcting the observed correlations due to various artefacts, empirical evidence has shown this to be the most accurate method [156,157].

As regards the limitations of the present meta-analysis, the main ones were related to the small number of criterion-related validity coefficients found. Estimating the population parameters based on small samples is simply less accurate than in a large-sized meta-analysis. Due to the low *K* found, a partial hierarchical breakdown had to be used instead of a full. Additionally, due to the low *K* found, the criterion-related validity of potentially different subcategories such as children (< 12 years) and adolescents (12–18 years) had to be examined together. Therefore, misleading results due to confounding and interaction effects might also be produced [16]. When a greater number of studies are accumulated, a large sized meta-analysis with more specific subcategories and a full hierarchical analysis approach should be carried out.

Another potential limitation could be related to the statistical metric. The correlation coefficient is a measure of *relationship* rather than *agreement* which it might be also highly influenced by the range of individual measurements [158]. The performance scores of the field tests (i.e. distance, time or speed) and the criterion measure (i.e. VO_2_max) are expressed in two different units and, therefore, logically an agreement statistical approach could not be performed. To solve this methodological limitation, another kind of validity such as the cross-validity or criterion-referenced validity could be followed instead [159]. However, these approaches assess a different kind of validity and they were not the scope of the present meta-analysis. For instance, although the criterion-referenced validity could be useful for screening if individuals are or not in a “health fitness zone”, the criterion-related validity is more appropriate for other purposes such as analyzing the effects of an intervention program. Future research studies should examine the cross-validity and criterion-referenced validity of the walk/run field tests. The validity of other field tests such as the walk or step tests should be also examined.

As regards the potential influence of the range of individuals’ measurement on criterion-related validity, the results of Spearman’s rank order correlations between the criterion-related validity coefficients and the standard deviation of the VO_2_max did not show any statistically significant association (*p* > 0.05), except for the 2 miles walk/run test (*r* = 0.71, *p* = 0.009). Therefore, in the present meta-analysis the empirical outcomes showed that the criterion-related validity of the most walk/run tests was not biased by the variability of the sample measurements.

Another limitation could be related to the criterion measure. Although only primary studies in which the criterion measure used the VO_2_max relative to body mass during a laboratory incremental test to exhaustion were selected, researchers employed different equipment, ergometers and protocols, as well as criteria to determine VO_2_max. It must be also highlighted the fact that the peak oxygen uptake (VO_2_peak) was used interchangeably with VO_2_max. Although the VO_2_peak simply refers to the highest value of oxygen uptake attained in a particular exercise test, due to the fact that the tests in the primary studies were maximal it can be reasonably sure that values were the highest value of oxygen uptake that is deemed attainable by individuals, i.e. the VO_2_max [160]. Therefore, the criterion measure of cardiorespiratory fitness should be standardized and reexamined [161].

Finally, coding some study features was problematic due to different reasons. Some study features simply could not be coded because the authors did not report them. Although authors were contacted by email and/or ResearchGate, many of them did not reply and the particular study feature had to be omitted. Also noting that many studies were published several years ago and, therefore, no contact email address and/or ResearchGate profile was found. Moreover, because the level of VO_2_max was classified based on the average scores, some individuals with low VO_2_max could be classified as high VO_2_max and vice versa. Finally, although there could be other potentially moderating features such as physical activity levels, coding for them was not possible because it was not reported in most of the studies.

## Conclusions

The overall criterion-related validity of the distance- and time-based walk/run field tests for estimating cardiorespiratory fitness ranged from low to moderate. The results of the present meta-analysis also showed that sex, age and VO_2_max levels do not seem to affect their criterion-related validity. The 1.5 mile and 12 min walk/run tests seem to be the best option of distance- and time-based field tests, respectively. Meanwhile performing longer walk/run tests could be an unnecessary extra time and effort, shorter tests showed poorer results of criterion-related validity.

When the evaluation of individual’s VO_2_max attained during a laboratory test is not feasible, the 1.5 mile and 12 min walk/run tests represent useful alternatives to estimate cardiorespiratory fitness. As in the assessment with any physical fitness field test, evaluators must be aware that the performance score of the walk/run field tests is simply an estimation and not a direct measure of cardiorespiratory fitness. Additionally, due to the relatively low number of *r* values found and that criterion-related validity values of walk/run field tests within most categories were still heterogeneous, the results of the present study should be considered with caution and firmer conclusions should await the accumulation of a larger number of studies.

## Supporting Information

S1 Appendix Syntaxes used in the present study for the search with the electronic bibliographic databases. (DOC)

S1 Fig Results of the cumulative meta-analyses by year of publication for criterion-related validity coefficients (*r*_p_) across the walk/run field tests: (a) 5,000 m walk/run test; (b) 2 miles walk/run test; (c) 3,000 m walk/run test; (d) 1.5 mile walk/run test; (e) 1 mile walk/run test; (f) 12 min walk/run test; and (g) 9 min walk/run test. (DOC)

S1 Table Summary of the included studies examining the criterion-related validity of walk/run field tests for estimating cardiorespiratory fitness. (DOC)

S2 Table Checklist. (DOC)
