# Supplementary material for: Criterion-Related Validity of the Distance- and Time-Based Walk/Run Field Tests for Estimating Cardiorespiratory Fitness: A Systematic Review and Meta-Analysis
Source: PLoS One. 2016 Mar 17;11(3):e0151671. doi: 10.1371/journal.pone.0151671 (PMC4795745; doi:10.1371/journal.pone.0151671)
Supplement: S1 Appendix — (DOC) [file pone.0151671.s001.doc]

**S1 Appendix. Syntaxes used in the present study for the search with the electronic bibliographic databases.**

**5,000 m walk/run test. Syntax 1**

("5 kilomet* run*" OR "5 kilomet* walk*" OR "5 kilomet* jog*" OR "5 kilomet* test*" OR "5 kilomet* track" OR "5 kilomet* endurance" OR "5 kilomet* aerobic" OR "5 kilomet* field" OR "5 kilomet* distance" OR "5 kilomet* performance" OR "5-kilomet* run*" OR "5-kilomet* walk*" OR "5-kilomet* jog*" OR "5-kilomet* test*" OR "5-kilomet* track" OR "5-kilomet* endurance" OR "5-kilomet* aerobic" OR "5-kilomet* field" OR "5-kilomet* distance" OR "5-kilomet* performance" OR "5 km run*" OR "5 km walk*" OR "5 km jog*" OR "5 km test*" OR "5 km track" OR "5 km endurance" OR "5 km aerobic" OR "5 km field" OR "5 km distance" OR "5 km performance" OR "5-km run*" OR "5-km walk*" OR "5-km jog*" OR "5-km test*" OR "5-km track" OR "5-km endurance" OR "5-km aerobic" OR "5-km field" OR "5-km distance" OR "5-km performance" OR "5km run*" OR "5km walk*" OR "5km jog*" OR "5km test*" OR "5km track" OR "5km endurance" OR "5km aerobic" OR "5km field" OR "5km distance" OR "5km performance" OR "five kilomet* run*" OR "five kilomet* walk*" OR "five kilomet* jog*" OR "five kilomet* test*" OR "five kilomet* track" OR "five kilomet* endurance" OR "five kilomet* aerobic" OR "five kilomet* field" OR "five kilomet* distance" OR "five kilomet* performance" OR "five-kilomet* run*" OR "five-kilomet* walk*" OR "five-kilomet* jog*" OR "five-kilomet* test*" OR "five-kilomet* track" OR "five-kilomet* endurance" OR "five-kilomet* aerobic" OR "five-kilomet* field" OR "five-kilomet* distance" OR "five-kilomet* performance" OR "five km run*" OR "five km walk*" OR "five km jog*" OR "five km test*" OR "five km track" OR "five km endurance" OR "five km aerobic" OR "five km field" OR "five km distance" OR "five km performance" OR "five-km run*" OR "five-km walk*" OR "five-km jog*" OR "five-km test*" OR "five-km track" OR "five-km endurance" OR "five-km aerobic" OR "five-km field" OR "five-km distance" OR "five-km performance") AND (valid* OR relat* OR correlat* OR compar* OR associat* OR regression OR estimat* OR determinat* OR predict* OR equation* OR VO2* OR "oxygen uptake" OR "oxygen intake" OR "consumption of oxygen" OR "oxygen consumption" OR aerobic OR cardiovascular OR cardiorespiratory OR fitness OR "gold standard" OR "criterion measur*" OR formula* OR "indirect calorimetry" OR "laboratory test*" OR treadmill OR "cycle ergometer" OR "bicycle ergometer" OR "direct measur*" OR "direct method*")

**5,000 m walk/run test. Syntax 2**

("5,000 met* run*" OR "5,000 met* walk*" OR "5,000 met* jog*" OR "5,000 met* test*" OR "5,000 met* track" OR "5,000 met* endurance" OR "5,000 met* aerobic" OR "5,000 met* field" OR "5,000 met* distance" OR "5,000 met* performance" OR "5,000-met* run*" OR "5,000-met* walk*" OR "5,000-met* jog*" OR "5,000-met* test*" OR "5,000-met* track" OR "5,000-met* endurance" OR "5,000-met* aerobic" OR "5,000-met* field" OR "5,000-met* distance" OR "5,000-met* performance" OR "5,000 m run*" OR "5,000 m walk*" OR "5,000 m jog*" OR "5,000 m test*" OR "5,000 m track" OR "5,000 m endurance" OR "5,000 m aerobic" OR "5,000 m field" OR "5,000 m distance" OR "5,000 m performance" OR "5,000-m run*" OR "5,000-m walk*" OR "5,000-m jog*" OR "5,000-m test*" OR "5,000-m track" OR "5,000-m endurance" OR "5,000-m aerobic" OR "5,000-m field" OR "5,000-m distance" OR "5,000-m performance" OR "5000 met* run*" OR "5000 met* walk*" OR "5000 met* jog*" OR "5000 met* test*" OR "5000 met* track" OR "5000 met* endurance" OR "5000 met* aerobic" OR "5000 met* field" OR "5000 met* distance" OR "5000 met* performance" OR "5000-met* run*" OR "5000-met* walk*" OR "5000-met* jog*" OR "5000-met* test*" OR "5000-met* track" OR "5000-met* endurance" OR "5000-met* aerobic" OR "5000-met* field" OR "5000-met* distance" OR "5000-met* performance" OR "5000 m run*" OR "5000 m walk*" OR "5000 m jog*" OR "5000 m test*" OR "5000 m track" OR "5000 m endurance" OR "5000 m aerobic" OR "5000 m field" OR "5000 m distance" OR "5000 m performance" OR "5000-m run*" OR "5000-m walk*" OR "5000-m jog*" OR "5000-m test*" OR "5000-m track" OR "5000-m endurance" OR "5000-m aerobic" OR "5000-m field" OR "5000-m distance" OR "5000-m performance" OR "5,000m run*" OR "5,000m walk*" OR "5,000m jog*" OR "5,000m test*" OR "5,000m track" OR "5,000m endurance" OR "5,000m aerobic" OR "5,000m field" OR "5,000m distance" OR "5,000m performance" OR "5000m run*" OR "5000m walk*" OR "5000m jog*" OR "5000m test*" OR "5000m track" OR "5000m endurance" OR "5000m aerobic" OR "5000m field" OR "5000m distance" OR "5000m performance") AND (valid* OR relat* OR correlat* OR compar* OR associat* OR regression OR estimat* OR determinat* OR predict* OR equation* OR VO2* OR "oxygen uptake" OR "oxygen intake" OR "consumption of oxygen" OR "oxygen consumption" OR aerobic OR cardiovascular OR cardiorespiratory OR fitness OR "gold standard" OR "criterion measur*" OR formula* OR "indirect calorimetry" OR "laboratory test*" OR treadmill OR "cycle ergometer" OR "bicycle ergometer" OR "direct measur*" OR "direct method*")

**3 miles walk/run test. Syntax 1**

("3 mile* run*" OR "3 mile* walk*" OR "3 mile* jog*" OR "3 mile* test*" OR "3 mile* track" OR "3 mile* endurance" OR "3 mile* aerobic" OR "3 mile* field" OR "3 mile* distance" OR "3 mile* performance" OR "3-mile run*" OR "3-mile walk*" OR "3-mile jog*" OR "3-mile test*" OR "3-mile track" OR "3-mile endurance" OR "3-mile aerobic" OR "3-mile field" OR "3-mile distance" OR "3-mile performance" OR "three mile* run*" OR "three mile* walk*" OR "three mile* jog*" OR "three mile* test*" OR "three mile* track" OR "three mile* endurance" OR "three mile* aerobic" OR "three mile* field" OR "three mile* distance" OR "three mile* performance" OR "three-mile run*" OR "three-mile walk*" OR "three-mile jog*" OR "three-mile test*" OR "three-mile track" OR "three-mile endurance" OR "three-mile aerobic" OR "three-mile field" OR "three-mile distance" OR "three-mile performance" OR "3 mi run*" OR "3 mi walk*" OR "3 mi jog*" OR "3 mi test*" OR "3 mi track" OR "3 mi endurance" OR "3 mi aerobic" OR "3 mi field" OR "3 mi distance" OR "3 mi performance" OR "3-mi run*" OR "3-mi walk*" OR "3-mi jog*" OR "3-mi test*" OR "3-mi track" OR "3-mi endurance" OR "3-mi aerobic" OR "3-mi field" OR "3-mi distance" OR "3-mi performance" OR "three mi run*" OR "three mi walk*" OR "three mi jog*" OR "three mi test*" OR "three mi track" OR "three mi endurance" OR "three mi aerobic" OR "three mi field" OR "three mi distance" OR "three mi performance" OR "three-mi run*" OR "three-mi walk*" OR "three-mi jog*" OR "three-mi test*" OR "three-mi track" OR "three-mi endurance" OR "three-mi aerobic" OR "three-mi field" OR "three-mi distance" OR "three-mi performance" OR "3mi run*" OR "3mi walk*" OR "3mi jog*" OR "3mi test*" OR "3mi track" OR "3mi endurance" OR "3mi aerobic" OR "3mi field" OR "3mi distance" OR "3mi performance") AND (valid* OR relat* OR correlat* OR compar* OR associat* OR regression OR estimat* OR determinat* OR predict* OR equation* OR VO2* OR "oxygen uptake" OR "oxygen intake" OR "consumption of oxygen" OR "oxygen consumption" OR aerobic OR cardiovascular OR cardiorespiratory OR fitness OR "gold standard" OR "criterion measur*" OR formula* OR "indirect calorimetry" OR "laboratory test*" OR treadmill OR "cycle ergometer" OR "bicycle ergometer" OR "direct measur*" OR "direct method*")

**3 miles walk/run test. Syntax 2**

("4.8 kilomet* run*" OR "4.8 kilomet* walk*" OR "4.8 kilomet* jog*" OR "4.8 kilomet* test*" OR "4.8 kilomet* track" OR "4.8 kilomet* endurance" OR "4.8 kilomet* aerobic" OR "4.8 kilomet* field" OR "4.8 kilomet* distance" OR "4.8 kilomet* performance" OR "4.8-kilomet* run*" OR "4.8-kilomet* walk*" OR "4.8-kilomet* jog*" OR "4.8-kilomet* test*" OR "4.8-kilomet* track" OR "4.8-kilomet* endurance" OR "4.8-kilomet* aerobic" OR "4.8-kilomet* field" OR "4.8-kilomet* distance" OR "4.8-kilomet* performance" OR "4.8 km run*" OR "4.8 km walk*" OR "4.8 km jog*" OR "4.8 km test*" OR "4.8 km track" OR "4.8 km endurance" OR "4.8 km aerobic" OR "4.8 km field" OR "4.8 km distance" OR "4.8 km performance" OR "4.8-km run*" OR "4.8-km walk*" OR "4.8-km jog*" OR "4.8-km test*" OR "4.8-km track" OR "4.8-km endurance" OR "4.8-km aerobic" OR "4.8-km field" OR "4.8-km distance" OR "4.8-km performance" OR "4.8km run*" OR "4.8km walk*" OR "4.8km jog*" OR "4.8km test*" OR "4.8km track" OR "4.8km endurance" OR "4.8km aerobic" OR "4.8km field" OR "4.8km distance" OR "4.8km performance") AND (valid* OR relat* OR correlat* OR compar* OR associat* OR regression OR estimat* OR determinat* OR predict* OR equation* OR VO2* OR "oxygen uptake" OR "oxygen intake" OR "consumption of oxygen" OR "oxygen consumption" OR aerobic OR cardiovascular OR cardiorespiratory OR fitness OR "gold standard" OR "criterion measur*" OR formula* OR "indirect calorimetry" OR "laboratory test*" OR treadmill OR "cycle ergometer" OR "bicycle ergometer" OR "direct measur*" OR "direct method*")

**3 miles walk/run test. Syntax 3**

("4,800 met* run*" OR "4,800 met* walk*" OR "4,800 met* jog*" OR "4,800 met* test*" OR "4,800 met* track" OR "4,800 met* endurance" OR "4,800 met* aerobic" OR "4,800 met* field" OR "4,800 met* distance" OR "4,800 met* performance" OR "4,800-met* run*" OR "4,800-met* walk*" OR "4,800-met* jog*" OR "4,800-met* test*" OR "4,800-met* track" OR "4,800-met* endurance" OR "4,800-met* aerobic" OR "4,800-met* field" OR "4,800-met* distance" OR "4,800-met* performance" OR "4,800 m run*" OR "4,800 m walk*" OR "4,800 m jog*" OR "4,800 m test*" OR "4,800 m track" OR "4,800 m endurance" OR "4,800 m aerobic" OR "4,800 m field" OR "4,800 m distance" OR "4,800 m performance" OR "4,800-m run*" OR "4,800-m walk*" OR "4,800-m jog*" OR "4,800-m test*" OR "4,800-m track" OR "4,800-m endurance" OR "4,800-m aerobic" OR "4,800-m field" OR "4,800-m distance" OR "4,800-m performance" OR "4800 met* run*" OR "4800 met* walk*" OR "4800 met* jog*" OR "4800 met* test*" OR "4800 met* track" OR "4800 met* endurance" OR "4800 met* aerobic" OR "4800 met* field" OR "4800 met* distance" OR "4800 met* performance" OR "4800-met* run*" OR "4800-met* walk*" OR "4800-met* jog*" OR "4800-met* test*" OR "4800-met* track" OR "4800-met* endurance" OR "4800-met* aerobic" OR "4800-met* field" OR "4800-met* distance" OR "4800-met* performance" OR "4800 m run*" OR "4800 m walk*" OR "4800 m jog*" OR "4800 m test*" OR "4800 m track" OR "4800 m endurance" OR "4800 m aerobic" OR "4800 m field" OR "4800 m distance" OR "4800 m performance" OR "4800-m run*" OR "4800-m walk*" OR "4800-m jog*" OR "4800-m test*" OR "4800-m track" OR "4800-m endurance" OR "4800-m aerobic" OR "4800-m field" OR "4800-m distance" OR "4800-m performance" OR "4,800m run*" OR "4,800m walk*" OR "4,800m jog*" OR "4,800m test*" OR "4,800m track" OR "4,800m endurance" OR "4,800m aerobic" OR "4,800m field" OR "4,800m distance" OR "4,800m performance" OR "4800m run*" OR "4800m walk*" OR "4800m jog*" OR "4800m test*" OR "4800m track" OR "4800m endurance" OR "4800m aerobic" OR "4800m field" OR "4800m distance" OR "4800m performance") AND (valid* OR relat* OR correlat* OR compar* OR associat* OR regression OR estimat* OR determinat* OR predict* OR equation* OR VO2* OR "oxygen uptake" OR "oxygen intake" OR "consumption of oxygen" OR "oxygen consumption" OR aerobic OR cardiovascular OR cardiorespiratory OR fitness OR "gold standard" OR "criterion measur*" OR formula* OR "indirect calorimetry" OR "laboratory test*" OR treadmill OR "cycle ergometer" OR "bicycle ergometer" OR "direct measur*" OR "direct method*")

**2 miles walk/run test. Syntax 1**

("2 mile* run*" OR "2 mile* walk*" OR "2 mile* jog*" OR "2 mile* test*" OR "2 mile* track" OR "2 mile* endurance" OR "2 mile* aerobic" OR "2 mile* field" OR "2 mile* distance" OR "2 mile* performance" OR "2-mile run*" OR "2-mile walk*" OR "2-mile jog*" OR "2-mile test*" OR "2-mile track" OR "2-mile endurance" OR "2-mile aerobic" OR "2-mile field" OR "2-mile distance" OR "2-mile performance" OR "two mile* run*" OR "two mile* walk*" OR "two mile* jog*" OR "two mile* test*" OR "two mile* track" OR "two mile* endurance" OR "two mile* aerobic" OR "two mile* field" OR "two mile* distance" OR "two mile* performance" OR "two-mile run*" OR "two-mile walk*" OR "two-mile jog*" OR "two-mile test*" OR "two-mile track" OR "two-mile endurance" OR "two-mile aerobic" OR "two-mile field" OR "two-mile distance" OR "two-mile performance" OR "2 mi run*" OR "2 mi walk*" OR "2 mi jog*" OR "2 mi test*" OR "2 mi track" OR "2 mi endurance" OR "2 mi aerobic" OR "2 mi field" OR "2 mi distance" OR "2 mi performance" OR "2-mi run*" OR "2-mi walk*" OR "2-mi jog*" OR "2-mi test*" OR "2-mi track" OR "2-mi endurance" OR "2-mi aerobic" OR "2-mi field" OR "2-mi distance" OR "2-mi performance" OR "two mi run*" OR "two mi walk*" OR "two mi jog*" OR "two mi test*" OR "two mi track" OR "two mi endurance" OR "two mi aerobic" OR "two mi field" OR "two mi distance" OR "two mi performance" OR "two-mi run*" OR "two-mi walk*" OR "two-mi jog*" OR "two-mi test*" OR "two-mi track" OR "two-mi endurance" OR "two-mi aerobic" OR "two-mi field" OR "two-mi distance" OR "two-mi performance" OR "2mi run*" OR "2mi walk*" OR "2mi jog*" OR "2mi test*" OR "2mi track" OR "2mi endurance" OR "2mi aerobic" OR "2mi field" OR "2mi distance" OR "2mi performance") AND (valid* OR relat* OR correlat* OR compar* OR associat* OR regression OR estimat* OR determinat* OR predict* OR equation* OR VO2* OR "oxygen uptake" OR "oxygen intake" OR "consumption of oxygen" OR "oxygen consumption" OR aerobic OR cardiovascular OR cardiorespiratory OR fitness OR "gold standard" OR "criterion measur*" OR formula* OR "indirect calorimetry" OR "laboratory test*" OR treadmill OR "cycle ergometer" OR "bicycle ergometer" OR "direct measur*" OR "direct method*")

**2 miles walk/run test. Syntax 2**

("3.2 kilomet* run*" OR "3.2 kilomet* walk*" OR "3.2 kilomet* jog*" OR "3.2 kilomet* test*" OR "3.2 kilomet* track" OR "3.2 kilomet* endurance" OR "3.2 kilomet* aerobic" OR "3.2 kilomet* field" OR "3.2 kilomet* distance" OR "3.2 kilomet* performance" OR "3.2-kilomet* run*" OR "3.2-kilomet* walk*" OR "3.2-kilomet* jog*" OR "3.2-kilomet* test*" OR "3.2-kilomet* track" OR "3.2-kilomet* endurance" OR "3.2-kilomet* aerobic" OR "3.2-kilomet* field" OR "3.2-kilomet* distance" OR "3.2-kilomet* performance" OR "3.2 km run*" OR "3.2 km walk*" OR "3.2 km jog*" OR "3.2 km test*" OR "3.2 km track" OR "3.2 km endurance" OR "3.2 km aerobic" OR "3.2 km field" OR "3.2 km distance" OR "3.2 km performance" OR "3.2-km run*" OR "3.2-km walk*" OR "3.2-km jog*" OR "3.2-km test*" OR "3.2-km track" OR "3.2-km endurance" OR "3.2-km aerobic" OR "3.2-km field" OR "3.2-km distance" OR "3.2-km performance" OR "3.2km run*" OR "3.2km walk*" OR "3.2km jog*" OR "3.2km test*" OR "3.2km track" OR "3.2km endurance" OR "3.2km aerobic" OR "3.2km field" OR "3.2km distance" OR "3.2km performance") AND (valid* OR relat* OR correlat* OR compar* OR associat* OR regression OR estimat* OR determinat* OR predict* OR equation* OR VO2* OR "oxygen uptake" OR "oxygen intake" OR "consumption of oxygen" OR "oxygen consumption" OR aerobic OR cardiovascular OR cardiorespiratory OR fitness OR "gold standard" OR "criterion measur*" OR formula* OR "indirect calorimetry" OR "laboratory test*" OR treadmill OR "cycle ergometer" OR "bicycle ergometer" OR "direct measur*" OR "direct method*")

**2 miles walk/run test. Syntax 3**

("3,200 met* run*" OR "3,200 met* walk*" OR "3,200 met* jog*" OR "3,200 met* test*" OR "3,200 met* track" OR "3,200 met* endurance" OR "3,200 met* aerobic" OR "3,200 met* field" OR "3,200 met* distance" OR "3,200 met* performance" OR "3,200-met* run*" OR "3,200-met* walk*" OR "3,200-met* jog*" OR "3,200-met* test*" OR "3,200-met* track" OR "3,200-met* endurance" OR "3,200-met* aerobic" OR "3,200-met* field" OR "3,200-met* distance" OR "3,200-met* performance" OR "3,200 m run*" OR "3,200 m walk*" OR "3,200 m jog*" OR "3,200 m test*" OR "3,200 m track" OR "3,200 m endurance" OR "3,200 m aerobic" OR "3,200 m field" OR "3,200 m distance" OR "3,200 m performance" OR "3,200-m run*" OR "3,200-m walk*" OR "3,200-m jog*" OR "3,200-m test*" OR "3,200-m track" OR "3,200-m endurance" OR "3,200-m aerobic" OR "3,200-m field" OR "3,200-m distance" OR "3,200-m performance" OR "3200 met* run*" OR "3200 met* walk*" OR "3200 met* jog*" OR "3200 met* test*" OR "3200 met* track" OR "3200 met* endurance" OR "3200 met* aerobic" OR "3200 met* field" OR "3200 met* distance" OR "3200 met* performance" OR "3200-met* run*" OR "3200-met* walk*" OR "3200-met* jog*" OR "3200-met* test*" OR "3200-met* track" OR "3200-met* endurance" OR "3200-met* aerobic" OR "3200-met* field" OR "3200-met* distance" OR "3200-met* performance" OR "3200 m run*" OR "3200 m walk*" OR "3200 m jog*" OR "3200 m test*" OR "3200 m track" OR "3200 m endurance" OR "3200 m aerobic" OR "3200 m field" OR "3200 m distance" OR "3200 m performance" OR "3200-m run*" OR "3200-m walk*" OR "3200-m jog*" OR "3200-m test*" OR "3200-m track" OR "3200-m endurance" OR "3200-m aerobic" OR "3200-m field" OR "3200-m distance" OR "3200-m performance" OR "3,200m run*" OR "3,200m walk*" OR "3,200m jog*" OR "3,200m test*" OR "3,200m track" OR "3,200m endurance" OR "3,200m aerobic" OR "3,200m field" OR "3,200m distance" OR "3,200m performance" OR "3200m run*" OR "3200m walk*" OR "3200m jog*" OR "3200m test*" OR "3200m track" OR "3200m endurance" OR "3200m aerobic" OR "3200m field" OR "3200m distance" OR "3200m performance") AND (valid* OR relat* OR correlat* OR compar* OR associat* OR regression OR estimat* OR determinat* OR predict* OR equation* OR VO2* OR "oxygen uptake" OR "oxygen intake" OR "consumption of oxygen" OR "oxygen consumption" OR aerobic OR cardiovascular OR cardiorespiratory OR fitness OR "gold standard" OR "criterion measur*" OR formula* OR "indirect calorimetry" OR "laboratory test*" OR treadmill OR "cycle ergometer" OR "bicycle ergometer" OR "direct measur*" OR "direct method*")

**3,000 m walk/run test. Syntax 1**

("3 kilomet* run*" OR "3 kilomet* walk*" OR "3 kilomet* jog*" OR "3 kilomet* test*" OR "3 kilomet* track" OR "3 kilomet* endurance" OR "3 kilomet* aerobic" OR "3 kilomet* field" OR "3 kilomet* distance" OR "3 kilomet* performance" OR "3-kilomet* run*" OR "3-kilomet* walk*" OR "3-kilomet* jog*" OR "3-kilomet* test*" OR "3-kilomet* track" OR "3-kilomet* endurance" OR "3-kilomet* aerobic" OR "3-kilomet* field" OR "3-kilomet* distance" OR "3-kilomet* performance" OR "3 km run*" OR "3 km walk*" OR "3 km jog*" OR "3 km test*" OR "3 km track" OR "3 km endurance" OR "3 km aerobic" OR "3 km field" OR "3 km distance" OR "3 km performance" OR "3-km run*" OR "3-km walk*" OR "3-km jog*" OR "3-km test*" OR "3-km track" OR "3-km endurance" OR "3-km aerobic" OR "3-km field" OR "3-km distance" OR "3-km performance" OR "3km run*" OR "3km walk*" OR "3km jog*" OR "3km test*" OR "3km track" OR "3km endurance" OR "3km aerobic" OR "3km field" OR "3km distance" OR "3km performance" OR "three kilomet* run*" OR "three kilomet* walk*" OR "three kilomet* jog*" OR "three kilomet* test*" OR "three kilomet* track" OR "three kilomet* endurance" OR "three kilomet* aerobic" OR "three kilomet* field" OR "three kilomet* distance" OR "three kilomet* performance" OR "three-kilomet* run*" OR "three-kilomet* walk*" OR "three-kilomet* jog*" OR "three-kilomet* test*" OR "three-kilomet* track" OR "three-kilomet* endurance" OR "three-kilomet* aerobic" OR "three-kilomet* field" OR "three-kilomet* distance" OR "three-kilomet* performance" OR "three km run*" OR "three km walk*" OR "three km jog*" OR "three km test*" OR "three km track" OR "three km endurance" OR "three km aerobic" OR "three km field" OR "three km distance" OR "three km performance" OR "three-km run*" OR "three-km walk*" OR "three-km jog*" OR "three-km test*" OR "three-km track" OR "three-km endurance" OR "three-km aerobic" OR "three-km field" OR "three-km distance" OR "three-km performance") AND (valid* OR relat* OR correlat* OR compar* OR associat* OR regression OR estimat* OR determinat* OR predict* OR equation* OR VO2* OR "oxygen uptake" OR "oxygen intake" OR "consumption of oxygen" OR "oxygen consumption" OR aerobic OR cardiovascular OR cardiorespiratory OR fitness OR "gold standard" OR "criterion measur*" OR formula* OR "indirect calorimetry" OR "laboratory test*" OR treadmill OR "cycle ergometer" OR "bicycle ergometer" OR "direct measur*" OR "direct method*")

**3,000 m walk/run test. Syntax 2**

("3,000 met* run*" OR "3,000 met* walk*" OR "3,000 met* jog*" OR "3,000 met* test*" OR "3,000 met* track" OR "3,000 met* endurance" OR "3,000 met* aerobic" OR "3,000 met* field" OR "3,000 met* distance" OR "3,000 met* performance" OR "3,000-met* run*" OR "3,000-met* walk*" OR "3,000-met* jog*" OR "3,000-met* test*" OR "3,000-met* track" OR "3,000-met* endurance" OR "3,000-met* aerobic" OR "3,000-met* field" OR "3,000-met* distance" OR "3,000-met* performance" OR "3,000 m run*" OR "3,000 m walk*" OR "3,000 m jog*" OR "3,000 m test*" OR "3,000 m track" OR "3,000 m endurance" OR "3,000 m aerobic" OR "3,000 m field" OR "3,000 m distance" OR "3,000 m performance" OR "3,000-m run*" OR "3,000-m walk*" OR "3,000-m jog*" OR "3,000-m test*" OR "3,000-m track" OR "3,000-m endurance" OR "3,000-m aerobic" OR "3,000-m field" OR "3,000-m distance" OR "3,000-m performance" OR "3000 met* run*" OR "3000 met* walk*" OR "3000 met* jog*" OR "3000 met* test*" OR "3000 met* track" OR "3000 met* endurance" OR "3000 met* aerobic" OR "3000 met* field" OR "3000 met* distance" OR "3000 met* performance" OR "3000-met* run*" OR "3000-met* walk*" OR "3000-met* jog*" OR "3000-met* test*" OR "3000-met* track" OR "3000-met* endurance" OR "3000-met* aerobic" OR "3000-met* field" OR "3000-met* distance" OR "3000-met* performance" OR "3000 m run*" OR "3000 m walk*" OR "3000 m jog*" OR "3000 m test*" OR "3000 m track" OR "3000 m endurance" OR "3000 m aerobic" OR "3000 m field" OR "3000 m distance" OR "3000 m performance" OR "3000-m run*" OR "3000-m walk*" OR "3000-m jog*" OR "3000-m test*" OR "3000-m track" OR "3000-m endurance" OR "3000-m aerobic" OR "3000-m field" OR "3000-m distance" OR "3000-m performance" OR "3,000m run*" OR "3,000m walk*" OR "3,000m jog*" OR "3,000m test*" OR "3,000m track" OR "3,000m endurance" OR "3,000m aerobic" OR "3,000m field" OR "3,000m distance" OR "3,000m performance" OR "3000m run*" OR "3000m walk*" OR "3000m jog*" OR "3000m test*" OR "3000m track" OR "3000m endurance" OR "3000m aerobic" OR "3000m field" OR "3000m distance" OR "3000m performance") AND (valid* OR relat* OR correlat* OR compar* OR associat* OR regression OR estimat* OR determinat* OR predict* OR equation* OR VO2* OR "oxygen uptake" OR "oxygen intake" OR "consumption of oxygen" OR "oxygen consumption" OR aerobic OR cardiovascular OR cardiorespiratory OR fitness OR "gold standard" OR "criterion measur*" OR formula* OR "indirect calorimetry" OR "laboratory test*" OR treadmill OR "cycle ergometer" OR "bicycle ergometer" OR "direct measur*" OR "direct method*")

**1.5 mile walk/run test. Syntax 1**

("1.5 mile* run*" OR "1.5 mile* walk*" OR "1.5 mile* jog*" OR "1.5 mile* test*" OR "1.5 mile* track" OR "1.5 mile* endurance" OR "1.5 mile* aerobic" OR "1.5 mile* field" OR "1.5 mile* distance" OR "1.5 mile* performance" OR "1.5-mile run*" OR "1.5-mile walk*" OR "1.5-mile jog*" OR "1.5-mile test*" OR "1.5-mile track" OR "1.5-mile endurance" OR "1.5-mile aerobic" OR "1.5-mile field" OR "1.5-mile distance" OR "1.5-mile performance" OR "one and half mile* run*" OR "one and half mile* walk*" OR "one and half mile* jog*" OR "one and half mile* test*" OR "one and half mile* track" OR "one and half mile* endurance" OR "one and half mile* aerobic" OR "one and half mile* field" OR "one and half mile* distance" OR "one and half mile* performance" OR "one-and-half mile* run*" OR "one-and-half mile* walk*" OR "one-and-half mile* jog*" OR "one-and-half mile* test*" OR "one-and-half mile* track" OR "one-and-half mile* endurance" OR "one-and-half mile* aerobic" OR "one-and-half mile* field" OR "one-and-half mile* distance" OR "one-and-half mile* performance" OR "one-and-half-mile run*" OR "one-and-half-mile walk*" OR "one-and-half-mile jog*" OR "one-and-half-mile test*" OR "one-and-half-mile track" OR "one-and-half-mile endurance" OR "one-and-half-mile aerobic" OR "one-and-half-mile field" OR "one-and-half-mile distance" OR "one-and-half-mile performance" OR "1.5 mi run*" OR "1.5 mi walk*" OR "1.5 mi jog*" OR "1.5 mi test*" OR "1.5 mi track" OR "1.5 mi endurance" OR "1.5 mi aerobic" OR "1.5 mi field" OR "1.5 mi distance" OR "1.5 mi performance" OR "1.5-mi run*" OR "1.5-mi walk*" OR "1.5-mi jog*" OR "1.5-mi test*" OR "1.5-mi track" OR "1.5-mi endurance" OR "1.5-mi aerobic" OR "1.5-mi field" OR "1.5-mi distance" OR "1.5-mi performance" OR "one and half mi run*" OR "one and half mi walk*" OR "one and half mi jog*" OR "one and half mi test*" OR "one and half mi track" OR "one and half mi endurance" OR "one and half mi aerobic" OR "one and half mi field" OR "one and half mi distance" OR "one and half mi performance" OR "one-and-half mi run*" OR "one-and-half mi walk*" OR "one-and-half mi jog*" OR "one-and-half mi test*" OR "one-and-half mi track" OR "one-and-half mi endurance" OR "one-and-half mi aerobic" OR "one-and-half mi field" OR "one-and-half mi distance" OR "one-and-half mi performance" OR "one-and-half-mi run*" OR "one-and-half-mi walk*" OR "one-and-half-mi jog*" OR "one-and-half-mi test*" OR "one-and-half-mi track" OR "one-and-half-mi endurance" OR "one-and-half-mi aerobic" OR "one-and-half-mi field" OR "one-and-half-mi distance" OR "one-and-half-mi performance" OR "1.5mi run*" OR "1.5mi walk*" OR "1.5mi jog*" OR "1.5mi test*" OR "1.5mi track" OR "1.5mi endurance" OR "1.5mi aerobic" OR "1.5mi field" OR "1.5mi distance" OR "1.5mi performance") AND (valid* OR relat* OR correlat* OR compar* OR associat* OR regression OR estimat* OR determinat* OR predict* OR equation* OR VO2* OR "oxygen uptake" OR "oxygen intake" OR "consumption of oxygen" OR "oxygen consumption" OR aerobic OR cardiovascular OR cardiorespiratory OR fitness OR "gold standard" OR "criterion measur*" OR formula* OR "indirect calorimetry" OR "laboratory test*" OR treadmill OR "cycle ergometer" OR "bicycle ergometer" OR "direct measur*" OR "direct method*")

**1.5 mile walk/run test. Syntax 2**

("2.4 kilomet* run*" OR "2.4 kilomet* walk*" OR "2.4 kilomet* jog*" OR "2.4 kilomet* test*" OR "2.4 kilomet* track" OR "2.4 kilomet* endurance" OR "2.4 kilomet* aerobic" OR "2.4 kilomet* field" OR "2.4 kilomet* distance" OR "2.4 kilomet* performance" OR "2.4-kilomet* run*" OR "2.4-kilomet* walk*" OR "2.4-kilomet* jog*" OR "2.4-kilomet* test*" OR "2.4-kilomet* track" OR "2.4-kilomet* endurance" OR "2.4-kilomet* aerobic" OR "2.4-kilomet* field" OR "2.4-kilomet* distance" OR "2.4-kilomet* performance" OR "2.4 km run*" OR "2.4 km walk*" OR "2.4 km jog*" OR "2.4 km test*" OR "2.4 km track" OR "2.4 km endurance" OR "2.4 km aerobic" OR "2.4 km field" OR "2.4 km distance" OR "2.4 km performance" OR "2.4-km run*" OR "2.4-km walk*" OR "2.4-km jog*" OR "2.4-km test*" OR "2.4-km track" OR "2.4-km endurance" OR "2.4-km aerobic" OR "2.4-km field" OR "2.4-km distance" OR "2.4-km performance" OR "2.4km run*" OR "2.4km walk*" OR "2.4km jog*" OR "2.4km test*" OR "2.4km track" OR "2.4km endurance" OR "2.4km aerobic" OR "2.4km field" OR "2.4km distance" OR "2.4km performance") AND (valid* OR relat* OR correlat* OR compar* OR associat* OR regression OR estimat* OR determinat* OR predict* OR equation* OR VO2* OR "oxygen uptake" OR "oxygen intake" OR "consumption of oxygen" OR "oxygen consumption" OR aerobic OR cardiovascular OR cardiorespiratory OR fitness OR "gold standard" OR "criterion measur*" OR formula* OR "indirect calorimetry" OR "laboratory test*" OR treadmill OR "cycle ergometer" OR "bicycle ergometer" OR "direct measur*" OR "direct method*")

**1.5 mile walk/run test. Syntax 3**

("2,400 met* run*" OR "2,400 met* walk*" OR "2,400 met* jog*" OR "2,400 met* test*" OR "2,400 met* track" OR "2,400 met* endurance" OR "2,400 met* aerobic" OR "2,400 met* field" OR "2,400 met* distance" OR "2,400 met* performance" OR "2,400-met* run*" OR "2,400-met* walk*" OR "2,400-met* jog*" OR "2,400-met* test*" OR "2,400-met* track" OR "2,400-met* endurance" OR "2,400-met* aerobic" OR "2,400-met* field" OR "2,400-met* distance" OR "2,400-met* performance" OR "2,400 m run*" OR "2,400 m walk*" OR "2,400 m jog*" OR "2,400 m test*" OR "2,400 m track" OR "2,400 m endurance" OR "2,400 m aerobic" OR "2,400 m field" OR "2,400 m distance" OR "2,400 m performance" OR "2,400-m run*" OR "2,400-m walk*" OR "2,400-m jog*" OR "2,400-m test*" OR "2,400-m track" OR "2,400-m endurance" OR "2,400-m aerobic" OR "2,400-m field" OR "2,400-m distance" OR "2,400-m performance" OR "2400 met* run*" OR "2400 met* walk*" OR "2400 met* jog*" OR "2400 met* test*" OR "2400 met* track" OR "2400 met* endurance" OR "2400 met* aerobic" OR "2400 met* field" OR "2400 met* distance" OR "2400 met* performance" OR "2400-met* run*" OR "2400-met* walk*" OR "2400-met* jog*" OR "2400-met* test*" OR "2400-met* track" OR "2400-met* endurance" OR "2400-met* aerobic" OR "2400-met* field" OR "2400-met* distance" OR "2400-met* performance" OR "2400 m run*" OR "2400 m walk*" OR "2400 m jog*" OR "2400 m test*" OR "2400 m track" OR "2400 m endurance" OR "2400 m aerobic" OR "2400 m field" OR "2400 m distance" OR "2400 m performance" OR "2400-m run*" OR "2400-m walk*" OR "2400-m jog*" OR "2400-m test*" OR "2400-m track" OR "2400-m endurance" OR "2400-m aerobic" OR "2400-m field" OR "2400-m distance" OR "2400-m performance" OR "2,400m run*" OR "2,400m walk*" OR "2,400m jog*" OR "2,400m test*" OR "2,400m track" OR "2,400m endurance" OR "2,400m aerobic" OR "2,400m field" OR "2,400m distance" OR "2,400m performance" OR "2400m run*" OR "2400m walk*" OR "2400m jog*" OR "2400m test*" OR "2400m track" OR "2400m endurance" OR "2400m aerobic" OR "2400m field" OR "2400m distance" OR "2400m performance") AND (valid* OR relat* OR correlat* OR compar* OR associat* OR regression OR estimat* OR determinat* OR predict* OR equation* OR VO2* OR "oxygen uptake" OR "oxygen intake" OR "consumption of oxygen" OR "oxygen consumption" OR aerobic OR cardiovascular OR cardiorespiratory OR fitness OR "gold standard" OR "criterion measur*" OR formula* OR "indirect calorimetry" OR "laboratory test*" OR treadmill OR "cycle ergometer" OR "bicycle ergometer" OR "direct measur*" OR "direct method*")

**1 mile walk/run test. Syntax 1**

("1 mile* run*" OR "1 mile* walk*" OR "1 mile* jog*" OR "1 mile* test*" OR "1 mile* track" OR "1 mile* endurance" OR "1 mile* aerobic" OR "1 mile* field" OR "1 mile* distance" OR "1 mile* performance" OR "1-mile run*" OR "1-mile walk*" OR "1-mile jog*" OR "1-mile test*" OR "1-mile track" OR "1-mile endurance" OR "1-mile aerobic" OR "1-mile field" OR "1-mile distance" OR "1-mile performance" OR "one mile* run*" OR "one mile* walk*" OR "one mile* jog*" OR "one mile* test*" OR "one mile* track" OR "one mile* endurance" OR "one mile* aerobic" OR "one mile* field" OR "one mile* distance" OR "one mile* performance" OR "one-mile run*" OR "one-mile walk*" OR "one-mile jog*" OR "one-mile test*" OR "one-mile track" OR "one-mile endurance" OR "one-mile aerobic" OR "one-mile field" OR "one-mile distance" OR "one-mile performance" OR "1 mi run*" OR "1 mi walk*" OR "1 mi jog*" OR "1 mi test*" OR "1 mi track" OR "1 mi endurance" OR "1 mi aerobic" OR "1 mi field" OR "1 mi distance" OR "1 mi performance" OR "1-mi run*" OR "1-mi walk*" OR "1-mi jog*" OR "1-mi test*" OR "1-mi track" OR "1-mi endurance" OR "1-mi aerobic" OR "1-mi field" OR "1-mi distance" OR "1-mi performance" OR "one mi run*" OR "one mi walk*" OR "one mi jog*" OR "one mi test*" OR "one mi track" OR "one mi endurance" OR "one mi aerobic" OR "one mi field" OR "one mi distance" OR "one mi performance" OR "one-mi run*" OR "one-mi walk*" OR "one-mi jog*" OR "one-mi test*" OR "one-mi track" OR "one-mi endurance" OR "one-mi aerobic" OR "one-mi field" OR "one-mi distance" OR "one-mi performance" OR "1mi run*" OR "1mi walk*" OR "1mi jog*" OR "1mi test*" OR "1mi track" OR "1mi endurance" OR "1mi aerobic" OR "1mi field" OR "1mi distance" OR "1mi performance") AND (valid* OR relat* OR correlat* OR compar* OR associat* OR regression OR estimat* OR determinat* OR predict* OR equation* OR VO2* OR "oxygen uptake" OR "oxygen intake" OR "consumption of oxygen" OR "oxygen consumption" OR aerobic OR cardiovascular OR cardiorespiratory OR fitness OR "gold standard" OR "criterion measur*" OR formula* OR "indirect calorimetry" OR "laboratory test*" OR treadmill OR "cycle ergometer" OR "bicycle ergometer" OR "direct measur*" OR "direct method*")

**1 mile walk/run test. Syntax 2**

("1.6 kilomet* run*" OR "1.6 kilomet* walk*" OR "1.6 kilomet* jog*" OR "1.6 kilomet* test*" OR "1.6 kilomet* track" OR "1.6 kilomet* endurance" OR "1.6 kilomet* aerobic" OR "1.6 kilomet* field" OR "1.6 kilomet* distance" OR "1.6 kilomet* performance" OR "1.6-kilomet* run*" OR "1.6-kilomet* walk*" OR "1.6-kilomet* jog*" OR "1.6-kilomet* test*" OR "1.6-kilomet* track" OR "1.6-kilomet* endurance" OR "1.6-kilomet* aerobic" OR "1.6-kilomet* field" OR "1.6-kilomet* distance" OR "1.6-kilomet* performance" OR "1.6 km run*" OR "1.6 km walk*" OR "1.6 km jog*" OR "1.6 km test*" OR "1.6 km track" OR "1.6 km endurance" OR "1.6 km aerobic" OR "1.6 km field" OR "1.6 km distance" OR "1.6 km performance" OR "1.6-km run*" OR "1.6-km walk*" OR "1.6-km jog*" OR "1.6-km test*" OR "1.6-km track" OR "1.6-km endurance" OR "1.6-km aerobic" OR "1.6-km field" OR "1.6-km distance" OR "1.6-km performance" OR "1.6km run*" OR "1.6km walk*" OR "1.6km jog*" OR "1.6km test*" OR "1.6km track" OR "1.6km endurance" OR "1.6km aerobic" OR "1.6km field" OR "1.6km distance" OR "1.6km performance") AND (valid* OR relat* OR correlat* OR compar* OR associat* OR regression OR estimat* OR determinat* OR predict* OR equation* OR VO2* OR "oxygen uptake" OR "oxygen intake" OR "consumption of oxygen" OR "oxygen consumption" OR aerobic OR cardiovascular OR cardiorespiratory OR fitness OR "gold standard" OR "criterion measur*" OR formula* OR "indirect calorimetry" OR "laboratory test*" OR treadmill OR "cycle ergometer" OR "bicycle ergometer" OR "direct measur*" OR "direct method*")

**1 mile walk/run test. Syntax 3**

("1,600 met* run*" OR "1,600 met* walk*" OR "1,600 met* jog*" OR "1,600 met* test*" OR "1,600 met* track" OR "1,600 met* endurance" OR "1,600 met* aerobic" OR "1,600 met* field" OR "1,600 met* distance" OR "1,600 met* performance" OR "1,600-met* run*" OR "1,600-met* walk*" OR "1,600-met* jog*" OR "1,600-met* test*" OR "1,600-met* track" OR "1,600-met* endurance" OR "1,600-met* aerobic" OR "1,600-met* field" OR "1,600-met* distance" OR "1,600-met* performance" OR "1,600 m run*" OR "1,600 m walk*" OR "1,600 m jog*" OR "1,600 m test*" OR "1,600 m track" OR "1,600 m endurance" OR "1,600 m aerobic" OR "1,600 m field" OR "1,600 m distance" OR "1,600 m performance" OR "1,600-m run*" OR "1,600-m walk*" OR "1,600-m jog*" OR "1,600-m test*" OR "1,600-m track" OR "1,600-m endurance" OR "1,600-m aerobic" OR "1,600-m field" OR "1,600-m distance" OR "1,600-m performance" OR "1600 met* run*" OR "1600 met* walk*" OR "1600 met* jog*" OR "1600 met* test*" OR "1600 met* track" OR "1600 met* endurance" OR "1600 met* aerobic" OR "1600 met* field" OR "1600 met* distance" OR "1600 met* performance" OR "1600-met* run*" OR "1600-met* walk*" OR "1600-met* jog*" OR "1600-met* test*" OR "1600-met* track" OR "1600-met* endurance" OR "1600-met* aerobic" OR "1600-met* field" OR "1600-met* distance" OR "1600-met* performance" OR "1600 m run*" OR "1600 m walk*" OR "1600 m jog*" OR "1600 m test*" OR "1600 m track" OR "1600 m endurance" OR "1600 m aerobic" OR "1600 m field" OR "1600 m distance" OR "1600 m performance" OR "1600-m run*" OR "1600-m walk*" OR "1600-m jog*" OR "1600-m test*" OR "1600-m track" OR "1600-m endurance" OR "1600-m aerobic" OR "1600-m field" OR "1600-m distance" OR "1600-m performance" OR "1,600m run*" OR "1,600m walk*" OR "1,600m jog*" OR "1,600m test*" OR "1,600m track" OR "1,600m endurance" OR "1,600m aerobic" OR "1,600m field" OR "1,600m distance" OR "1,600m performance" OR "1600m run*" OR "1600m walk*" OR "1600m jog*" OR "1600m test*" OR "1600m track" OR "1600m endurance" OR "1600m aerobic" OR "1600m field" OR "1600m distance" OR "1600m performance") AND (valid* OR relat* OR correlat* OR compar* OR associat* OR regression OR estimat* OR determinat* OR predict* OR equation* OR VO2* OR "oxygen uptake" OR "oxygen intake" OR "consumption of oxygen" OR "oxygen consumption" OR aerobic OR cardiovascular OR cardiorespiratory OR fitness OR "gold standard" OR "criterion measur*" OR formula* OR "indirect calorimetry" OR "laboratory test*" OR treadmill OR "cycle ergometer" OR "bicycle ergometer" OR "direct measur*" OR "direct method*")

**1,000 m walk/run test. Syntax 1**

("1 kilomet* run*" OR "1 kilomet* walk*" OR "1 kilomet* jog*" OR "1 kilomet* test*" OR "1 kilomet* track" OR "1 kilomet* endurance" OR "1 kilomet* aerobic" OR "1 kilomet* field" OR "1 kilomet* distance" OR "1 kilomet* performance" OR "1-kilomet* run*" OR "1-kilomet* walk*" OR "1-kilomet* jog*" OR "1-kilomet* test*" OR "1-kilomet* track" OR "1-kilomet* endurance" OR "1-kilomet* aerobic" OR "1-kilomet* field" OR "1-kilomet* distance" OR "1-kilomet* performance" OR "1 km run*" OR "1 km walk*" OR "1 km jog*" OR "1 km test*" OR "1 km track" OR "1 km endurance" OR "1 km aerobic" OR "1 km field" OR "1 km distance" OR "1 km performance" OR "1-km run*" OR "1-km walk*" OR "1-km jog*" OR "1-km test*" OR "1-km track" OR "1-km endurance" OR "1-km aerobic" OR "1-km field" OR "1-km distance" OR "1-km performance" OR "1km run*" OR "1km walk*" OR "1km jog*" OR "1km test*" OR "1km track" OR "1km endurance" OR "1km aerobic" OR "1km field" OR "1km distance" OR "1km performance" OR "one kilomet* run*" OR "one kilomet* walk*" OR "one kilomet* jog*" OR "one kilomet* test*" OR "one kilomet* track" OR "one kilomet* endurance" OR "one kilomet* aerobic" OR "one kilomet* field" OR "one kilomet* distance" OR "one kilomet* performance" OR "one-kilomet* run*" OR "one-kilomet* walk*" OR "one-kilomet* jog*" OR "one-kilomet* test*" OR "one-kilomet* track" OR "one-kilomet* endurance" OR "one-kilomet* aerobic" OR "one-kilomet* field" OR "one-kilomet* distance" OR "one-kilomet* performance" OR "one km run*" OR "one km walk*" OR "one km jog*" OR "one km test*" OR "one km track" OR "one km endurance" OR "one km aerobic" OR "one km field" OR "one km distance" OR "one km performance" OR "one-km run*" OR "one-km walk*" OR "one-km jog*" OR "one-km test*" OR "one-km track" OR "one-km endurance" OR "one-km aerobic" OR "one-km field" OR "one-km distance" OR "one-km performance") AND (valid* OR relat* OR correlat* OR compar* OR associat* OR regression OR estimat* OR determinat* OR predict* OR equation* OR VO2* OR "oxygen uptake" OR "oxygen intake" OR "consumption of oxygen" OR "oxygen consumption" OR aerobic OR cardiovascular OR cardiorespiratory OR fitness OR "gold standard" OR "criterion measur*" OR formula* OR "indirect calorimetry" OR "laboratory test*" OR treadmill OR "cycle ergometer" OR "bicycle ergometer" OR "direct measur*" OR "direct method*")

**1,000 m walk/run test. Syntax 2**

("1,000 met* run*" OR "1,000 met* walk*" OR "1,000 met* jog*" OR "1,000 met* test*" OR "1,000 met* track" OR "1,000 met* endurance" OR "1,000 met* aerobic" OR "1,000 met* field" OR "1,000 met* distance" OR "1,000 met* performance" OR "1,000-met* run*" OR "1,000-met* walk*" OR "1,000-met* jog*" OR "1,000-met* test*" OR "1,000-met* track" OR "1,000-met* endurance" OR "1,000-met* aerobic" OR "1,000-met* field" OR "1,000-met* distance" OR "1,000-met* performance" OR "1,000 m run*" OR "1,000 m walk*" OR "1,000 m jog*" OR "1,000 m test*" OR "1,000 m track" OR "1,000 m endurance" OR "1,000 m aerobic" OR "1,000 m field" OR "1,000 m distance" OR "1,000 m performance" OR "1,000-m run*" OR "1,000-m walk*" OR "1,000-m jog*" OR "1,000-m test*" OR "1,000-m track" OR "1,000-m endurance" OR "1,000-m aerobic" OR "1,000-m field" OR "1,000-m distance" OR "1,000-m performance" OR "1000 met* run*" OR "1000 met* walk*" OR "1000 met* jog*" OR "1000 met* test*" OR "1000 met* track" OR "1000 met* endurance" OR "1000 met* aerobic" OR "1000 met* field" OR "1000 met* distance" OR "1000 met* performance" OR "1000-met* run*" OR "1000-met* walk*" OR "1000-met* jog*" OR "1000-met* test*" OR "1000-met* track" OR "1000-met* endurance" OR "1000-met* aerobic" OR "1000-met* field" OR "1000-met* distance" OR "1000-met* performance" OR "1000 m run*" OR "1000 m walk*" OR "1000 m jog*" OR "1000 m test*" OR "1000 m track" OR "1000 m endurance" OR "1000 m aerobic" OR "1000 m field" OR "1000 m distance" OR "1000 m performance" OR "1000-m run*" OR "1000-m walk*" OR "1000-m jog*" OR "1000-m test*" OR "1000-m track" OR "1000-m endurance" OR "1000-m aerobic" OR "1000-m field" OR "1000-m distance" OR "1000-m performance" OR "1,000m run*" OR "1,000m walk*" OR "1,000m jog*" OR "1,000m test*" OR "1,000m track" OR "1,000m endurance" OR "1,000m aerobic" OR "1,000m field" OR "1,000m distance" OR "1,000m performance" OR "1000m run*" OR "1000m walk*" OR "1000m jog*" OR "1000m test*" OR "1000m track" OR "1000m endurance" OR "1000m aerobic" OR "1000m field" OR "1000m distance" OR "1000m performance") AND (valid* OR relat* OR correlat* OR compar* OR associat* OR regression OR estimat* OR determinat* OR predict* OR equation* OR VO2* OR "oxygen uptake" OR "oxygen intake" OR "consumption of oxygen" OR "oxygen consumption" OR aerobic OR cardiovascular OR cardiorespiratory OR fitness OR "gold standard" OR "criterion measur*" OR formula* OR "indirect calorimetry" OR "laboratory test*" OR treadmill OR "cycle ergometer" OR "bicycle ergometer" OR "direct measur*" OR "direct method*")

**½ mile walk/run test. Syntax 1**

("1/2 mile* run*" OR "1/2 mile* walk*" OR "1/2 mile* jog*" OR "1/2 mile* test*" OR "1/2 mile* track" OR "1/2 mile* endurance" OR "1/2 mile* aerobic" OR "1/2 mile* field" OR "1/2 mile* distance" OR "1/2 mile* performance" OR "1/2-mile run*" OR "1/2-mile walk*" OR "1/2-mile jog*" OR "1/2-mile test*" OR "1/2-mile track" OR "1/2-mile endurance" OR "1/2-mile aerobic" OR "1/2-mile field" OR "1/2-mile distance" OR "1/2-mile performance" OR "1/2 mi run*" OR "1/2 mi walk*" OR "1/2 mi jog*" OR "1/2 mi test*" OR "1/2 mi track" OR "1/2 mi endurance" OR "1/2 mi aerobic" OR "1/2 mi field" OR "1/2 mi distance" OR "1/2 mi performance" OR "1/2-mi run*" OR "1/2-mi walk*" OR "1/2-mi jog*" OR "1/2-mi test*" OR "1/2-mi track" OR "1/2-mi endurance" OR "1/2-mi aerobic" OR "1/2-mi field" OR "1/2-mi distance" OR "1/2-mi performance" OR "0.5 mile* run*" OR "0.5 mile* walk*" OR "0.5 mile* jog*" OR "0.5 mile* test*" OR "0.5 mile* track" OR "0.5 mile* endurance" OR "0.5 mile* aerobic" OR "0.5 mile* field" OR "0.5 mile* distance" OR "0.5 mile* performance" OR "0.5-mile run*" OR "0.5-mile walk*" OR "0.5-mile jog*" OR "0.5-mile test*" OR "0.5-mile track" OR "0.5-mile endurance" OR "0.5-mile aerobic" OR "0.5-mile field" OR "0.5-mile distance" OR "0.5-mile performance" OR "half mile* run*" OR "half mile* walk*" OR "half mile* jog*" OR "half mile* test*" OR "half mile* track" OR "half mile* endurance" OR "half mile* aerobic" OR "half mile* field" OR "half mile* distance" OR "half mile* performance" OR "half-mile run*" OR "half-mile walk*" OR "half-mile jog*" OR "half-mile test*" OR "half-mile track" OR "half-mile endurance" OR "half-mile aerobic" OR "half-mile field" OR "half-mile distance" OR "half-mile performance" OR "0.5 mi run*" OR "0.5 mi walk*" OR "0.5 mi jog*" OR "0.5 mi test*" OR "0.5 mi track" OR "0.5 mi endurance" OR "0.5 mi aerobic" OR "0.5 mi field" OR "0.5 mi distance" OR "0.5 mi performance" OR "0.5-mi run*" OR "0.5-mi walk*" OR "0.5-mi jog*" OR "0.5-mi test*" OR "0.5-mi track" OR "0.5-mi endurance" OR "0.5-mi aerobic" OR "0.5-mi field" OR "0.5-mi distance" OR "0.5-mi performance" OR "half mi run*" OR "half mi walk*" OR "half mi jog*" OR "half mi test*" OR "half mi track" OR "half mi endurance" OR "half mi aerobic" OR "half mi field" OR "half mi distance" OR "half mi performance" OR "half-mi run*" OR "half-mi walk*" OR "half-mi jog*" OR "half-mi test*" OR "half-mi track" OR "half-mi endurance" OR "half-mi aerobic" OR "half-mi field" OR "half-mi distance" OR "half-mi performance" OR "1/2mi run*" OR "1/2mi walk*" OR "1/2mi jog*" OR "1/2mi test*" OR "1/2mi track" OR "1/2mi endurance" OR "1/2mi aerobic" OR "1/2mi field" OR "1/2mi distance" OR "1/2mi performance" OR "0.5mi run*" OR "0.5mi walk*" OR "0.5mi jog*" OR "0.5mi test*" OR "0.5mi track" OR "0.5mi endurance" OR "0.5mi aerobic" OR "0.5mi field" OR "0.5mi distance" OR "0.5mi performance") AND (valid* OR relat* OR correlat* OR compar* OR associat* OR regression OR estimat* OR determinat* OR predict* OR equation* OR VO2* OR "oxygen uptake" OR "oxygen intake" OR "consumption of oxygen" OR "oxygen consumption" OR aerobic OR cardiovascular OR cardiorespiratory OR fitness OR "gold standard" OR "criterion measur*" OR formula* OR "indirect calorimetry" OR "laboratory test*" OR treadmill OR "cycle ergometer" OR "bicycle ergometer" OR "direct measur*" OR "direct method*")

**½ mile walk/run test. Syntax 2**

("800 met* run*" OR "800 met* walk*" OR "800 met* jog*" OR "800 met* test*" OR "800 met* track" OR "800 met* endurance" OR "800 met* aerobic" OR "800 met* field" OR "800 met* distance" OR "800 met* performance" OR "800-met* run*" OR "800-met* walk*" OR "800-met* jog*" OR "800-met* test*" OR "800-met* track" OR "800-met* endurance" OR "800-met* aerobic" OR "800-met* field" OR "800-met* distance" OR "800-met* performance" OR "800 m run*" OR "800 m walk*" OR "800 m jog*" OR "800 m test*" OR "800 m track" OR "800 m endurance" OR "800 m aerobic" OR "800 m field" OR "800 m distance" OR "800 m performance" OR "800-m run*" OR "800-m walk*" OR "800-m jog*" OR "800-m test*" OR "800-m track" OR "800-m endurance" OR "800-m aerobic" OR "800-m field" OR "800-m distance" OR "800-m performance" OR "800m run*" OR "800m walk*" OR "800m jog*" OR "800m test*" OR "800m track" OR "800m endurance" OR "800m aerobic" OR "800m field" OR "800m distance" OR "800m performance") AND (valid* OR relat* OR correlat* OR compar* OR associat* OR regression OR estimat* OR determinat* OR predict* OR equation* OR VO2* OR "oxygen uptake" OR "oxygen intake" OR "consumption of oxygen" OR "oxygen consumption" OR aerobic OR cardiovascular OR cardiorespiratory OR fitness OR "gold standard" OR "criterion measur*" OR formula* OR "indirect calorimetry" OR "laboratory test*" OR treadmill OR "cycle ergometer" OR "bicycle ergometer" OR "direct measur*" OR "direct method*")

**½ mile walk/run test. Syntax 3**

("880 yard* run*" OR "880 yard* walk*" OR "880 yard* jog*" OR "880 yard* test*" OR "880 yard* track" OR "880 yard* endurance" OR "880 yard* aerobic" OR "880 yard* field" OR "880 yard* distance" OR "880 yard* performance" OR "880-yard* run*" OR "880-yard* walk*" OR "880-yard* jog*" OR "880-yard* test*" OR "880-yard* track" OR "880-yard* endurance" OR "880-yard* aerobic" OR "880-yard* field" OR "880-yard* distance" OR "880-yard* performance" OR "880 yd run*" OR "880 yd walk*" OR "880 yd jog*" OR "880 yd test*" OR "880 yd track" OR "880 yd endurance" OR "880 yd aerobic" OR "880 yd field" OR "880 yd distance" OR "880 yd performance" OR "880-yd run*" OR "880-yd walk*" OR "880-yd jog*" OR "880-yd test*" OR "880-yd track" OR "880-yd endurance" OR "880-yd aerobic" OR "880-yd field" OR "880-yd distance" OR "880-yd performance" OR "880yd run*" OR "880yd walk*" OR "880yd jog*" OR "880yd test*" OR "880yd track" OR "880yd endurance" OR "880yd aerobic" OR "880yd field" OR "880yd distance" OR "880yd performance") AND (valid* OR relat* OR correlat* OR compar* OR associat* OR regression OR estimat* OR determinat* OR predict* OR equation* OR VO2* OR "oxygen uptake" OR "oxygen intake" OR "consumption of oxygen" OR "oxygen consumption" OR aerobic OR cardiovascular OR cardiorespiratory OR fitness OR "gold standard" OR "criterion measur*" OR formula* OR "indirect calorimetry" OR "laboratory test*" OR treadmill OR "cycle ergometer" OR "bicycle ergometer" OR "direct measur*" OR "direct method*")

**600 m walk/run test. Syntax 1**

("600 met* run*" OR "600 met* walk*" OR "600 met* jog*" OR "600 met* test*" OR "600 met* track" OR "600 met* endurance" OR "600 met* aerobic" OR "600 met* field" OR "600 met* distance" OR "600 met* performance" OR "600-met* run*" OR "600-met* walk*" OR "600-met* jog*" OR "600-met* test*" OR "600-met* track" OR "600-met* endurance" OR "600-met* aerobic" OR "600-met* field" OR "600-met* distance" OR "600-met* performance" OR "600 m run*" OR "600 m walk*" OR "600 m jog*" OR "600 m test*" OR "600 m track" OR "600 m endurance" OR "600 m aerobic" OR "600 m field" OR "600 m distance" OR "600 m performance" OR "600-m run*" OR "600-m walk*" OR "600-m jog*" OR "600-m test*" OR "600-m track" OR "600-m endurance" OR "600-m aerobic" OR "600-m field" OR "600-m distance" OR "600-m performance" OR "600m run*" OR "600m walk*" OR "600m jog*" OR "600m test*" OR "600m track" OR "600m endurance" OR "600m aerobic" OR "600m field" OR "600m distance" OR "600m performance") AND (valid* OR relat* OR correlat* OR compar* OR associat* OR regression OR estimat* OR determinat* OR predict* OR equation* OR VO2* OR "oxygen uptake" OR "oxygen intake" OR "consumption of oxygen" OR "oxygen consumption" OR aerobic OR cardiovascular OR cardiorespiratory OR fitness OR "gold standard" OR "criterion measur*" OR formula* OR "indirect calorimetry" OR "laboratory test*" OR treadmill OR "cycle ergometer" OR "bicycle ergometer" OR "direct measur*" OR "direct method*")

**600 m walk/run test. Syntax 2**

("650 yard* run*" OR "650 yard* walk*" OR "650 yard* jog*" OR "650 yard* test*" OR "650 yard* track" OR "650 yard* endurance" OR "650 yard* aerobic" OR "650 yard* field" OR "650 yard* distance" OR "650 yard* performance" OR "650-yard* run*" OR "650-yard* walk*" OR "650-yard* jog*" OR "650-yard* test*" OR "650-yard* track" OR "650-yard* endurance" OR "650-yard* aerobic" OR "650-yard* field" OR "650-yard* distance" OR "650-yard* performance" OR "650 yd run*" OR "650 yd walk*" OR "650 yd jog*" OR "650 yd test*" OR "650 yd track" OR "650 yd endurance" OR "650 yd aerobic" OR "650 yd field" OR "650 yd distance" OR "650 yd performance" OR "650-yd run*" OR "650-yd walk*" OR "650-yd jog*" OR "650-yd test*" OR "650-yd track" OR "650-yd endurance" OR "650-yd aerobic" OR "650-yd field" OR "650-yd distance" OR "650-yd performance" OR "650yd run*" OR "650yd walk*" OR "650yd jog*" OR "650yd test*" OR "650yd track" OR "650yd endurance" OR "650yd aerobic" OR "650yd field" OR "650yd distance" OR "650yd performance") AND (valid* OR relat* OR correlat* OR compar* OR associat* OR regression OR estimat* OR determinat* OR predict* OR equation* OR VO2* OR "oxygen uptake" OR "oxygen intake" OR "consumption of oxygen" OR "oxygen consumption" OR aerobic OR cardiovascular OR cardiorespiratory OR fitness OR "gold standard" OR "criterion measur*" OR formula* OR "indirect calorimetry" OR "laboratory test*" OR treadmill OR "cycle ergometer" OR "bicycle ergometer" OR "direct measur*" OR "direct method*")

**600 yd walk/run test. Syntax 1**

("550 met* run*" OR "550 met* walk*" OR "550 met* jog*" OR "550 met* test*" OR "550 met* track" OR "550 met* endurance" OR "550 met* aerobic" OR "550 met* field" OR "550 met* distance" OR "550 met* performance" OR "550-met* run*" OR "550-met* walk*" OR "550-met* jog*" OR "550-met* test*" OR "550-met* track" OR "550-met* endurance" OR "550-met* aerobic" OR "550-met* field" OR "550-met* distance" OR "550-met* performance" OR "550 m run*" OR "550 m walk*" OR "550 m jog*" OR "550 m test*" OR "550 m track" OR "550 m endurance" OR "550 m aerobic" OR "550 m field" OR "550 m distance" OR "550 m performance" OR "550-m run*" OR "550-m walk*" OR "550-m jog*" OR "550-m test*" OR "550-m track" OR "550-m endurance" OR "550-m aerobic" OR "550-m field" OR "550-m distance" OR "550-m performance" OR "550m run*" OR "550m walk*" OR "550m jog*" OR "550m test*" OR "550m track" OR "550m endurance" OR "550m aerobic" OR "550m field" OR "550m distance" OR "550m performance") AND (valid* OR relat* OR correlat* OR compar* OR associat* OR regression OR estimat* OR determinat* OR predict* OR equation* OR VO2* OR "oxygen uptake" OR "oxygen intake" OR "consumption of oxygen" OR "oxygen consumption" OR aerobic OR cardiovascular OR cardiorespiratory OR fitness OR "gold standard" OR "criterion measur*" OR formula* OR "indirect calorimetry" OR "laboratory test*" OR treadmill OR "cycle ergometer" OR "bicycle ergometer" OR "direct measur*" OR "direct method*")

**600 yd walk/run test. Syntax 2**

("600 yard* run*" OR "600 yard* walk*" OR "600 yard* jog*" OR "600 yard* test*" OR "600 yard* track" OR "600 yard* endurance" OR "600 yard* aerobic" OR "600 yard* field" OR "600 yard* distance" OR "600 yard* performance" OR "600-yard* run*" OR "600-yard* walk*" OR "600-yard* jog*" OR "600-yard* test*" OR "600-yard* track" OR "600-yard* endurance" OR "600-yard* aerobic" OR "600-yard* field" OR "600-yard* distance" OR "600-yard* performance" OR "600 yd run*" OR "600 yd walk*" OR "600 yd jog*" OR "600 yd test*" OR "600 yd track" OR "600 yd endurance" OR "600 yd aerobic" OR "600 yd field" OR "600 yd distance" OR "600 yd performance" OR "600-yd run*" OR "600-yd walk*" OR "600-yd jog*" OR "600-yd test*" OR "600-yd track" OR "600-yd endurance" OR "600-yd aerobic" OR "600-yd field" OR "600-yd distance" OR "600-yd performance" OR "600yd run*" OR "600yd walk*" OR "600yd jog*" OR "600yd test*" OR "600yd track" OR "600yd endurance" OR "600yd aerobic" OR "600yd field" OR "600yd distance" OR "600yd performance") AND (valid* OR relat* OR correlat* OR compar* OR associat* OR regression OR estimat* OR determinat* OR predict* OR equation* OR VO2* OR "oxygen uptake" OR "oxygen intake" OR "consumption of oxygen" OR "oxygen consumption" OR aerobic OR cardiovascular OR cardiorespiratory OR fitness OR "gold standard" OR "criterion measur*" OR formula* OR "indirect calorimetry" OR "laboratory test*" OR treadmill OR "cycle ergometer" OR "bicycle ergometer" OR "direct measur*" OR "direct method*")

**¼ mile walk/run test. Syntax 1**

("1/4 mile* run*" OR "1/4 mile* walk*" OR "1/4 mile* jog*" OR "1/4 mile* test*" OR "1/4 mile* track" OR "1/4 mile* endurance" OR "1/4 mile* aerobic" OR "1/4 mile* field" OR "1/4 mile* distance" OR "1/4 mile* performance" OR "1/4-mile run*" OR "1/4-mile walk*" OR "1/4-mile jog*" OR "1/4-mile test*" OR "1/4-mile track" OR "1/4-mile endurance" OR "1/4-mile aerobic" OR "1/4-mile field" OR "1/4-mile distance" OR "1/4-mile performance" OR "1/4 mi run*" OR "1/4 mi walk*" OR "1/4 mi jog*" OR "1/4 mi test*" OR "1/4 mi track" OR "1/4 mi endurance" OR "1/4 mi aerobic" OR "1/4 mi field" OR "1/4 mi distance" OR "1/4 mi performance" OR "1/4-mi run*" OR "1/4-mi walk*" OR "1/4-mi jog*" OR "1/4-mi test*" OR "1/4-mi track" OR "1/4-mi endurance" OR "1/4-mi aerobic" OR "1/4-mi field" OR "1/4-mi distance" OR "1/4-mi performance" OR "0.25 mile* run*" OR "0.25 mile* walk*" OR "0.25 mile* jog*" OR "0.25 mile* test*" OR "0.25 mile* track" OR "0.25 mile* endurance" OR "0.25 mile* aerobic" OR "0.25 mile* field" OR "0.25 mile* distance" OR "0.25 mile* performance" OR "0.25-mile run*" OR "0.25-mile walk*" OR "0.25-mile jog*" OR "0.25-mile test*" OR "0.25-mile track" OR "0.25-mile endurance" OR "0.25-mile aerobic" OR "0.25-mile field" OR "0.25-mile distance" OR "0.25-mile performance" OR "quarter mile* run*" OR "quarter mile* walk*" OR "quarter mile* jog*" OR "quarter mile* test*" OR "quarter mile* track" OR "quarter mile* endurance" OR "quarter mile* aerobic" OR "quarter mile* field" OR "quarter mile* distance" OR "quarter mile* performance" OR "quarter-mile run*" OR "quarter-mile walk*" OR "quarter-mile jog*" OR "quarter-mile test*" OR "quarter-mile track" OR "quarter-mile endurance" OR "quarter-mile aerobic" OR "quarter-mile field" OR "quarter-mile distance" OR "quarter-mile performance" OR "0.25 mi run*" OR "0.25 mi walk*" OR "0.25 mi jog*" OR "0.25 mi test*" OR "0.25 mi track" OR "0.25 mi endurance" OR "0.25 mi aerobic" OR "0.25 mi field" OR "0.25 mi distance" OR "0.25 mi performance" OR "0.25-mi run*" OR "0.25-mi walk*" OR "0.25-mi jog*" OR "0.25-mi test*" OR "0.25-mi track" OR "0.25-mi endurance" OR "0.25-mi aerobic" OR "0.25-mi field" OR "0.25-mi distance" OR "0.25-mi performance" OR "quarter mi run*" OR "quarter mi walk*" OR "quarter mi jog*" OR "quarter mi test*" OR "quarter mi track" OR "quarter mi endurance" OR "quarter mi aerobic" OR "quarter mi field" OR "quarter mi distance" OR "quarter mi performance" OR "quarter-mi run*" OR "quarter-mi walk*" OR "quarter-mi jog*" OR "quarter-mi test*" OR "quarter-mi track" OR "quarter-mi endurance" OR "quarter-mi aerobic" OR "quarter-mi field" OR "quarter-mi distance" OR "quarter-mi performance" OR "1/4mi run*" OR "1/4mi walk*" OR "1/4mi jog*" OR "1/4mi test*" OR "1/4mi track" OR "1/4mi endurance" OR "1/4mi aerobic" OR "1/4mi field" OR "1/4mi distance" OR "1/4mi performance" OR "0.25mi run*" OR "0.25mi walk*" OR "0.25mi jog*" OR "0.25mi test*" OR "0.25mi track" OR "0.25mi endurance" OR "0.25mi aerobic" OR "0.25mi field" OR "0.25mi distance" OR "0.25mi performance") AND (valid* OR relat* OR correlat* OR compar* OR associat* OR regression OR estimat* OR determinat* OR predict* OR equation* OR VO2* OR "oxygen uptake" OR "oxygen intake" OR "consumption of oxygen" OR "oxygen consumption" OR aerobic OR cardiovascular OR cardiorespiratory OR fitness OR "gold standard" OR "criterion measur*" OR formula* OR "indirect calorimetry" OR "laboratory test*" OR treadmill OR "cycle ergometer" OR "bicycle ergometer" OR "direct measur*" OR "direct method*")

**¼ mile walk/run test. Syntax 2**

("400 met* run*" OR "400 met* walk*" OR "400 met* jog*" OR "400 met* test*" OR "400 met* track" OR "400 met* endurance" OR "400 met* aerobic" OR "400 met* field" OR "400 met* distance" OR "400 met* performance" OR "400-met* run*" OR "400-met* walk*" OR "400-met* jog*" OR "400-met* test*" OR "400-met* track" OR "400-met* endurance" OR "400-met* aerobic" OR "400-met* field" OR "400-met* distance" OR "400-met* performance" OR "400 m run*" OR "400 m walk*" OR "400 m jog*" OR "400 m test*" OR "400 m track" OR "400 m endurance" OR "400 m aerobic" OR "400 m field" OR "400 m distance" OR "400 m performance" OR "400-m run*" OR "400-m walk*" OR "400-m jog*" OR "400-m test*" OR "400-m track" OR "400-m endurance" OR "400-m aerobic" OR "400-m field" OR "400-m distance" OR "400-m performance" OR "400m run*" OR "400m walk*" OR "400m jog*" OR "400m test*" OR "400m track" OR "400m endurance" OR "400m aerobic" OR "400m field" OR "400m distance" OR "400m performance") AND (valid* OR relat* OR correlat* OR compar* OR associat* OR regression OR estimat* OR determinat* OR predict* OR equation* OR VO2* OR "oxygen uptake" OR "oxygen intake" OR "consumption of oxygen" OR "oxygen consumption" OR aerobic OR cardiovascular OR cardiorespiratory OR fitness OR "gold standard" OR "criterion measur*" OR formula* OR "indirect calorimetry" OR "laboratory test*" OR treadmill OR "cycle ergometer" OR "bicycle ergometer" OR "direct measur*" OR "direct method*")

**¼ mile walk/run test. Syntax 3**

("440 yard* run*" OR "440 yard* walk*" OR "440 yard* jog*" OR "440 yard* test*" OR "440 yard* track" OR "440 yard* endurance" OR "440 yard* aerobic" OR "440 yard* field" OR "440 yard* distance" OR "440 yard* performance" OR "440-yard* run*" OR "440-yard* walk*" OR "440-yard* jog*" OR "440-yard* test*" OR "440-yard* track" OR "440-yard* endurance" OR "440-yard* aerobic" OR "440-yard* field" OR "440-yard* distance" OR "440-yard* performance" OR "440 yd run*" OR "440 yd walk*" OR "440 yd jog*" OR "440 yd test*" OR "440 yd track" OR "440 yd endurance" OR "440 yd aerobic" OR "440 yd field" OR "440 yd distance" OR "440 yd performance" OR "440-yd run*" OR "440-yd walk*" OR "440-yd jog*" OR "440-yd test*" OR "440-yd track" OR "440-yd endurance" OR "440-yd aerobic" OR "440-yd field" OR "440-yd distance" OR "440-yd performance" OR "440yd run*" OR "440yd walk*" OR "440yd jog*" OR "440yd test*" OR "440yd track" OR "440yd endurance" OR "440yd aerobic" OR "440yd field" OR "440yd distance" OR "440yd performance") AND (valid* OR relat* OR correlat* OR compar* OR associat* OR regression OR estimat* OR determinat* OR predict* OR equation* OR VO2* OR "oxygen uptake" OR "oxygen intake" OR "consumption of oxygen" OR "oxygen consumption" OR aerobic OR cardiovascular OR cardiorespiratory OR fitness OR "gold standard" OR "criterion measur*" OR formula* OR "indirect calorimetry" OR "laboratory test*" OR treadmill OR "cycle ergometer" OR "bicycle ergometer" OR "direct measur*" OR "direct method*")

**15 min walk/run test. Syntax 1**

("Balke run*" OR "Balke walk*" OR "Balke jog*" OR "Balke test*" OR "Balke track" OR "Balke endurance" OR "Balke aerobic" OR "Balke field" OR "Balke time" OR "Balke performance" OR "Balke´s run*" OR "Balke´s walk*" OR "Balke´s jog*" OR "Balke´s test*" OR "Balke´s track" OR "Balke´s endurance" OR "Balke´s aerobic" OR "Balke´s field" OR "Balke´s time" OR "Balke´s performance" OR "Balkes run*" OR "Balkes walk*" OR "Balkes jog*" OR "Balkes test*" OR "Balkes track" OR "Balkes endurance" OR "Balkes aerobic" OR "Balkes field" OR "Balkes time" OR "Balkes performance" OR "15 minute* run*" OR "15 minute* walk*" OR "15 minute* jog*" OR "15 minute* test*" OR "15 minute* track" OR "15 minute* endurance" OR "15 minute* aerobic" OR "15 minute* field" OR "15 minute* time" OR "15 minute* performance" OR "15-minute run*" OR "15-minute walk*" OR "15-minute jog*" OR "15-minute test*" OR "15-minute track" OR "15-minute endurance" OR "15-minute aerobic" OR "15-minute field" OR "15-minute time" OR "15-minute performance" OR "fifteen minute* run*" OR "fifteen minute* walk*" OR "fifteen minute* jog*" OR "fifteen minute* test*" OR "fifteen minute* track" OR "fifteen minute* endurance" OR "fifteen minute* aerobic" OR "fifteen minute* field" OR "fifteen minute* time" OR "fifteen minute* performance" OR "fifteen-minute run*" OR "fifteen-minute walk*" OR "fifteen-minute jog*" OR "fifteen-minute test*" OR "fifteen-minute track" OR "fifteen-minute endurance" OR "fifteen-minute aerobic" OR "fifteen-minute field" OR "fifteen-minute time" OR "fifteen-minute performance" OR "15 min run*" OR "15 min walk*" OR "15 min jog*" OR "15 min test*" OR "15 min track" OR "15 min endurance" OR "15 min aerobic" OR "15 min field" OR "15 min time" OR "15 min performance" OR "15-min run*" OR "15-min walk*" OR "15-min jog*" OR "15-min test*" OR "15-min track" OR "15-min endurance" OR "15-min aerobic" OR "15-min field" OR "15-min time" OR "15-min performance" OR "fifteen min run*" OR "fifteen min walk*" OR "fifteen min jog*" OR "fifteen min test*" OR "fifteen min track" OR "fifteen min endurance" OR "fifteen min aerobic" OR "fifteen min field" OR "fifteen min time" OR "fifteen min performance" OR "fifteen-min run*" OR "fifteen-min walk*" OR "fifteen-min jog*" OR "fifteen-min test*" OR "fifteen-min track" OR "fifteen-min endurance" OR "fifteen-min aerobic" OR "fifteen-min field" OR "fifteen-min time" OR "fifteen-min performance" OR "15min run*" OR "15min walk*" OR "15min jog*" OR "15min test*" OR "15min track" OR "15min endurance" OR "15min aerobic" OR "15min field" OR "15min time" OR "15min performance") AND (valid* OR relat* OR correlat* OR compar* OR associat* OR regression OR estimat* OR determinat* OR predict* OR equation* OR VO2* OR "oxygen uptake" OR "oxygen intake" OR "consumption of oxygen" OR "oxygen consumption" OR aerobic OR cardiovascular OR cardiorespiratory OR fitness OR "gold standard" OR "criterion measur*" OR formula* OR "indirect calorimetry" OR "laboratory test*" OR treadmill OR "cycle ergometer" OR "bicycle ergometer" OR "direct measur*" OR "direct method*")

**12 min walk/run test. Syntax 1**

("Cooper run*" OR "Cooper walk*" OR "Cooper jog*" OR "Cooper test*" OR "Cooper track" OR "Cooper endurance" OR "Cooper aerobic" OR "Cooper field" OR "Cooper time" OR "Cooper performance" OR "Cooper´s run*" OR "Cooper´s walk*" OR "Cooper´s jog*" OR "Cooper´s test*" OR "Cooper´s track" OR "Cooper´s endurance" OR "Cooper´s aerobic" OR "Cooper´s field" OR "Cooper´s time" OR "Cooper´s performance" OR "Coopers run*" OR "Coopers walk*" OR "Coopers jog*" OR "Coopers test*" OR "Coopers track" OR "Coopers endurance" OR "Coopers aerobic" OR "Coopers field" OR "Coopers time" OR "Coopers performance" OR "12 minute* run*" OR "12 minute* walk*" OR "12 minute* jog*" OR "12 minute* test*" OR "12 minute* track" OR "12 minute* endurance" OR "12 minute* aerobic" OR "12 minute* field" OR "12 minute* time" OR "12 minute* performance" OR "12-minute run*" OR "12-minute walk*" OR "12-minute jog*" OR "12-minute test*" OR "12-minute track" OR "12-minute endurance" OR "12-minute aerobic" OR "12-minute field" OR "12-minute time" OR "12-minute performance" OR "twelve minute* run*" OR "twelve minute* walk*" OR "twelve minute* jog*" OR "twelve minute* test*" OR "twelve minute* track" OR "twelve minute* endurance" OR "twelve minute* aerobic" OR "twelve minute* field" OR "twelve minute* time" OR "twelve minute* performance" OR "twelve-minute run*" OR "twelve-minute walk*" OR "twelve-minute jog*" OR "twelve-minute test*" OR "twelve-minute track" OR "twelve-minute endurance" OR "twelve-minute aerobic" OR "twelve-minute field" OR "twelve-minute time" OR "twelve-minute performance" OR "12 min run*" OR "12 min walk*" OR "12 min jog*" OR "12 min test*" OR "12 min track" OR "12 min endurance" OR "12 min aerobic" OR "12 min field" OR "12 min time" OR "12 min performance" OR "12-min run*" OR "12-min walk*" OR "12-min jog*" OR "12-min test*" OR "12-min track" OR "12-min endurance" OR "12-min aerobic" OR "12-min field" OR "12-min time" OR "12-min performance" OR "twelve min run*" OR "twelve min walk*" OR "twelve min jog*" OR "twelve min test*" OR "twelve min track" OR "twelve min endurance" OR "twelve min aerobic" OR "twelve min field" OR "twelve min time" OR "twelve min performance" OR "twelve-min run*" OR "twelve-min walk*" OR "twelve-min jog*" OR "twelve-min test*" OR "twelve-min track" OR "twelve-min endurance" OR "twelve-min aerobic" OR "twelve-min field" OR "twelve-min time" OR "twelve-min performance" OR "12min run*" OR "12min walk*" OR "12min jog*" OR "12min test*" OR "12min track" OR "12min endurance" OR "12min aerobic" OR "12min field" OR "12min time" OR "12min performance") AND (valid* OR relat* OR correlat* OR compar* OR associat* OR regression OR estimat* OR determinat* OR predict* OR equation* OR VO2* OR "oxygen uptake" OR "oxygen intake" OR "consumption of oxygen" OR "oxygen consumption" OR aerobic OR cardiovascular OR cardiorespiratory OR fitness OR "gold standard" OR "criterion measur*" OR formula* OR "indirect calorimetry" OR "laboratory test*" OR treadmill OR "cycle ergometer" OR "bicycle ergometer" OR "direct measur*" OR "direct method*")

**9 min walk/run test. Syntax 1**

("9 minute* run*" OR "9 minute* walk*" OR "9 minute* jog*" OR "9 minute* test*" OR "9 minute* track" OR "9 minute* endurance" OR "9 minute* aerobic" OR "9 minute* field" OR "9 minute* time" OR "9 minute* performance" OR "9-minute run*" OR "9-minute walk*" OR "9-minute jog*" OR "9-minute test*" OR "9-minute track" OR "9-minute endurance" OR "9-minute aerobic" OR "9-minute field" OR "9-minute time" OR "9-minute performance" OR "nine minute* run*" OR "nine minute* walk*" OR "nine minute* jog*" OR "nine minute* test*" OR "nine minute* track" OR "nine minute* endurance" OR "nine minute* aerobic" OR "nine minute* field" OR "nine minute* time" OR "nine minute* performance" OR "nine-minute run*" OR "nine-minute walk*" OR "nine-minute jog*" OR "nine-minute test*" OR "nine-minute track" OR "nine-minute endurance" OR "nine-minute aerobic" OR "nine-minute field" OR "nine-minute time" OR "nine-minute performance" OR "9 min run*" OR "9 min walk*" OR "9 min jog*" OR "9 min test*" OR "9 min track" OR "9 min endurance" OR "9 min aerobic" OR "9 min field" OR "9 min time" OR "9 min performance" OR "9-min run*" OR "9-min walk*" OR "9-min jog*" OR "9-min test*" OR "9-min track" OR "9-min endurance" OR "9-min aerobic" OR "9-min field" OR "9-min time" OR "9-min performance" OR "nine min run*" OR "nine min walk*" OR "nine min jog*" OR "nine min test*" OR "nine min track" OR "nine min endurance" OR "nine min aerobic" OR "nine min field" OR "nine min time" OR "nine min performance" OR "nine-min run*" OR "nine-min walk*" OR "nine-min jog*" OR "nine-min test*" OR "nine-min track" OR "nine-min endurance" OR "nine-min aerobic" OR "nine-min field" OR "nine-min time" OR "nine-min performance" OR "9min run*" OR "9min walk*" OR "9min jog*" OR "9min test*" OR "9min track" OR "9min endurance" OR "9min aerobic" OR "9min field" OR "9min time" OR "9min performance") AND (valid* OR relat* OR correlat* OR compar* OR associat* OR regression OR estimat* OR determinat* OR predict* OR equation* OR VO2* OR "oxygen uptake" OR "oxygen intake" OR "consumption of oxygen" OR "oxygen consumption" OR aerobic OR cardiovascular OR cardiorespiratory OR fitness OR "gold standard" OR "criterion measur*" OR formula* OR "indirect calorimetry" OR "laboratory test*" OR treadmill OR "cycle ergometer" OR "bicycle ergometer" OR "direct measur*" OR "direct method*")

**6 min walk/run test. Syntax 1**

("6 minute* run*" OR "6 minute* walk run*" OR "6 minute* walk-run*" OR "6 minute* walk/run*" OR "6 minute* walk and run*" OR "6 minute* walk-and-run*" OR "6 minute* walk & run*" OR "6 minute* walking run*" OR "6 minute* walking-run*" OR "6 minute* walking/run*" OR "6 minute* walking and run*" OR "6 minute* walking-and-run*" OR "6 minute* walking & run*" OR "6 minute* walk jog*" OR "6 minute* walk-jog*" OR "6 minute* walk/jog*" OR "6 minute* walk and jog*" OR "6 minute* walk-and-jog*" OR "6 minute* walk & jog*" OR "6 minute* walking jog*" OR "6 minute* walking-jog*" OR "6 minute* walking/jog*" OR "6 minute* walking and jog*" OR "6 minute* walking-and-jog*" OR "6 minute* walking & jog*" OR "6 minute* jog*" OR "6 minute* test*" OR "6 minute* track" OR "6 minute* endurance" OR "6 minute* aerobic" OR "6 minute* field" OR "6 minute* time" OR "6 minute* performance" OR "6-minute run*" OR "6-minute walk run*" OR "6-minute walk-run*" OR "6-minute walk/run*" OR "6-minute walk and run*" OR "6-minute walk-and-run*" OR "6-minute walk & run*" OR "6-minute walking run*" OR "6-minute walking-run*" OR "6-minute walking/run*" OR "6-minute walking and run*" OR "6-minute walking-and-run*" OR "6-minute walking & run*" OR "6-minute walk jog*" OR "6-minute walk-jog*" OR "6-minute walk/jog*" OR "6-minute walk and jog*" OR "6-minute walk-and-jog*" OR "6-minute walk & jog*" OR "6-minute walking jog*" OR "6-minute walking-jog*" OR "6-minute walking/jog*" OR "6-minute walking and jog*" OR "6-minute walking-and-jog*" OR "6-minute walking & jog*" OR "6-minute jog*" OR "6-minute test*" OR "6-minute track" OR "6-minute endurance" OR "6-minute aerobic" OR "6-minute field" OR "6-minute time" OR "6-minute performance") AND (valid* OR relat* OR correlat* OR compar* OR associat* OR regression OR estimat* OR determinat* OR predict* OR equation* OR VO2* OR "oxygen uptake" OR "oxygen intake" OR "consumption of oxygen" OR "oxygen consumption" OR aerobic OR cardiovascular OR cardiorespiratory OR fitness OR "gold standard" OR "criterion measur*" OR formula* OR "indirect calorimetry" OR "laboratory test*" OR treadmill OR "cycle ergometer" OR "bicycle ergometer" OR "direct measur*" OR "direct method*")

**6 min walk/run test. Syntax 2**

("six minute* run*" OR "six minute* walk run*" OR "six minute* walk-run*" OR "six minute* walk/run*" OR "six minute* walk and run*" OR "six minute* walk-and-run*" OR "six minute* walk & run*" OR "six minute* walking run*" OR "six minute* walking-run*" OR "six minute* walking/run*" OR "six minute* walking and run*" OR "six minute* walking-and-run*" OR "six minute* walking & run*" OR "six minute* walk jog*" OR "six minute* walk-jog*" OR "six minute* walk/jog*" OR "six minute* walk and jog*" OR "six minute* walk-and-jog*" OR "six minute* walk & jog*" OR "six minute* walking jog*" OR "six minute* walking-jog*" OR "six minute* walking/jog*" OR "six minute* walking and jog*" OR "six minute* walking-and-jog*" OR "six minute* walking & jog*" OR "six minute* jog*" OR "six minute* test*" OR "six minute* track" OR "six minute* endurance" OR "six minute* aerobic" OR "six minute* field" OR "six minute* time" OR "six minute* performance" OR "six-minute run*" OR "six-minute walk run*" OR "six-minute walk-run*" OR "six-minute walk/run*" OR "six-minute walk and run*" OR "six-minute walk-and-run*" OR "six-minute walk & run*" OR "six-minute walking run*" OR "six-minute walking-run*" OR "six-minute walking/run*" OR "six-minute walking and run*" OR "six-minute walking-and-run*" OR "six-minute walking & run*" OR "six-minute walk jog*" OR "six-minute walk-jog*" OR "six-minute walk/jog*" OR "six-minute walk and jog*" OR "six-minute walk-and-jog*" OR "six-minute walk & jog*" OR "six-minute walking jog*" OR "six-minute walking-jog*" OR "six-minute walking/jog*" OR "six-minute walking and jog*" OR "six-minute walking-and-jog*" OR "six-minute walking & jog*" OR "six-minute jog*" OR "six-minute test*" OR "six-minute track" OR "six-minute endurance" OR "six-minute aerobic" OR "six-minute field" OR "six-minute time" OR "six-minute performance") AND (valid* OR relat* OR correlat* OR compar* OR associat* OR regression OR estimat* OR determinat* OR predict* OR equation* OR VO2* OR "oxygen uptake" OR "oxygen intake" OR "consumption of oxygen" OR "oxygen consumption" OR aerobic OR cardiovascular OR cardiorespiratory OR fitness OR "gold standard" OR "criterion measur*" OR formula* OR "indirect calorimetry" OR "laboratory test*" OR treadmill OR "cycle ergometer" OR "bicycle ergometer" OR "direct measur*" OR "direct method*")

**6 min walk/run test. Syntax 3**

("6 min run*" OR "6 min walk run*" OR "6 min walk-run*" OR "6 min walk/run*" OR "6 min walk and run*" OR "6 min walk-and-run*" OR "6 min walk & run*" OR "6 min walking run*" OR "6 min walking-run*" OR "6 min walking/run*" OR "6 min walking and run*" OR "6 min walking-and-run*" OR "6 min walking & run*" OR "6 min walk jog*" OR "6 min walk-jog*" OR "6 min walk/jog*" OR "6 min walk and jog*" OR "6 min walk-and-jog*" OR "6 min walk & jog*" OR "6 min walking jog*" OR "6 min walking-jog*" OR "6 min walking/jog*" OR "6 min walking and jog*" OR "6 min walking-and-jog*" OR "6 min walking & jog*" OR "6 min jog*" OR "6 min test*" OR "6 min track" OR "6 min endurance" OR "6 min aerobic" OR "6 min field" OR "6 min time" OR "6 min performance" OR "6-min run*" OR "6-min walk run*" OR "6-min walk-run*" OR "6-min walk/run*" OR "6-min walk and run*" OR "6-min walk-and-run*" OR "6-min walk & run*" OR "6-min walking run*" OR "6-min walking-run*" OR "6-min walking/run*" OR "6-min walking and run*" OR "6-min walking-and-run*" OR "6-min walking & run*" OR "6-min walk jog*" OR "6-min walk-jog*" OR "6-min walk/jog*" OR "6-min walk and jog*" OR "6-min walk-and-jog*" OR "6-min walk & jog*" OR "6-min walking jog*" OR "6-min walking-jog*" OR "6-min walking/jog*" OR "6-min walking and jog*" OR "6-min walking-and-jog*" OR "6-min walking & jog*" OR "6-min jog*" OR "6-min test*" OR "6-min track" OR "6-min endurance" OR "6-min aerobic" OR "6-min field" OR "6-min time" OR "6-min performance") AND (valid* OR relat* OR correlat* OR compar* OR associat* OR regression OR estimat* OR determinat* OR predict* OR equation* OR VO2* OR "oxygen uptake" OR "oxygen intake" OR "consumption of oxygen" OR "oxygen consumption" OR aerobic OR cardiovascular OR cardiorespiratory OR fitness OR "gold standard" OR "criterion measur*" OR formula* OR "indirect calorimetry" OR "laboratory test*" OR treadmill OR "cycle ergometer" OR "bicycle ergometer" OR "direct measur*" OR "direct method*")

**6 min walk/run test. Syntax 4**

("six min run*" OR "six min walk run*" OR "six min walk-run*" OR "six min walk/run*" OR "six min walk and run*" OR "six min walk-and-run*" OR "six min walk & run*" OR "six min walking run*" OR "six min walking-run*" OR "six min walking/run*" OR "six min walking and run*" OR "six min walking-and-run*" OR "six min walking & run*" OR "six min walk jog*" OR "six min walk-jog*" OR "six min walk/jog*" OR "six min walk and jog*" OR "six min walk-and-jog*" OR "six min walk & jog*" OR "six min walking jog*" OR "six min walking-jog*" OR "six min walking/jog*" OR "six min walking and jog*" OR "six min walking-and-jog*" OR "six min walking & jog*" OR "six min jog*" OR "six min test*" OR "six min track" OR "six min endurance" OR "six min aerobic" OR "six min field" OR "six min time" OR "six min performance" OR "six-min run*" OR "six-min walk run*" OR "six-min walk-run*" OR "six-min walk/run*" OR "six-min walk and run*" OR "six-min walk-and-run*" OR "six-min walk & run*" OR "six-min walking run*" OR "six-min walking-run*" OR "six-min walking/run*" OR "six-min walking and run*" OR "six-min walking-and-run*" OR "six-min walking & run*" OR "six-min walk jog*" OR "six-min walk-jog*" OR "six-min walk/jog*" OR "six-min walk and jog*" OR "six-min walk-and-jog*" OR "six-min walk & jog*" OR "six-min walking jog*" OR "six-min walking-jog*" OR "six-min walking/jog*" OR "six-min walking and jog*" OR "six-min walking-and-jog*" OR "six-min walking & jog*" OR "six-min jog*" OR "six-min test*" OR "six-min track" OR "six-min endurance" OR "six-min aerobic" OR "six-min field" OR "six-min time" OR "six-min performance" OR "6min run*" OR "6min walk*" OR "6min jog*" OR "6min test*" OR "6min track" OR "6min endurance" OR "6min aerobic" OR "6min field" OR "6min time" OR "6min performance") AND (valid* OR relat* OR correlat* OR compar* OR associat* OR regression OR estimat* OR determinat* OR predict* OR equation* OR VO2* OR "oxygen uptake" OR "oxygen intake" OR "consumption of oxygen" OR "oxygen consumption" OR aerobic OR cardiovascular OR cardiorespiratory OR fitness OR "gold standard" OR "criterion measur*" OR formula* OR "indirect calorimetry" OR "laboratory test*" OR treadmill OR "cycle ergometer" OR "bicycle ergometer" OR "direct measur*" OR "direct method*")
