# Supplementary material for: Criterion-Related Validity of the Distance- and Time-Based Walk/Run Field Tests for Estimating Cardiorespiratory Fitness: A Systematic Review and Meta-Analysis
Source: PLoS One. 2016 Mar 17;11(3):e0151671. doi: 10.1371/journal.pone.0151671 (PMC4795745; doi:10.1371/journal.pone.0151671)

**S1 Fig. Results of the cumulative meta-analyses by year of publication for criterion-related validity coefficients (*r*_p_) across the walk/run field tests: (a) 5,000 m walk/run test; (b) 2 miles walk/run test; (c) 3,000 m walk/run test; (d) 1.5 mile walk/run test; (e) 1 mile walk/run test; (f) 12 min walk/run test; and (g) 9 min walk/run test.**

**
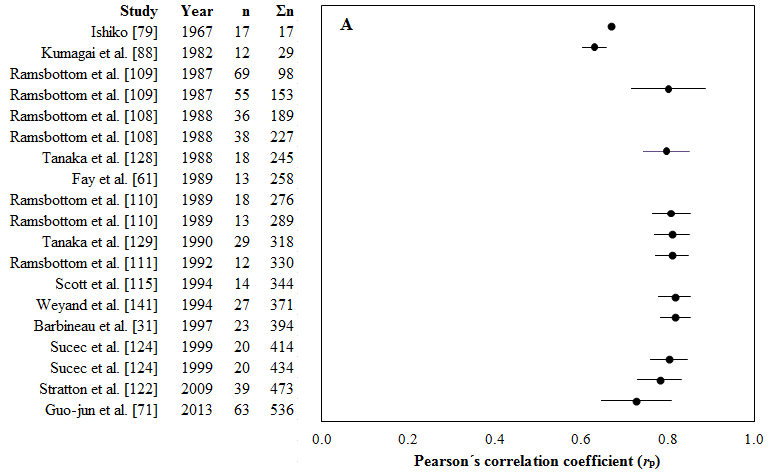
**


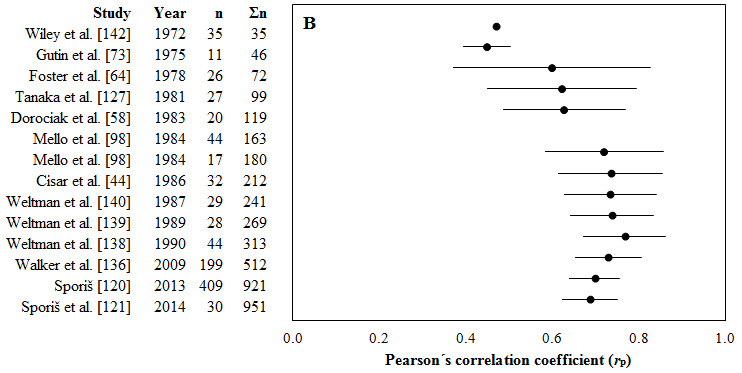


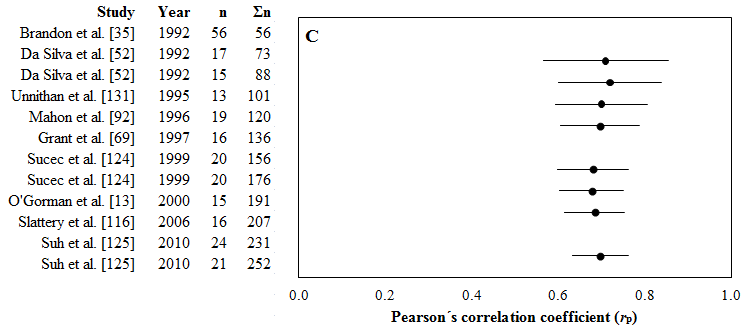


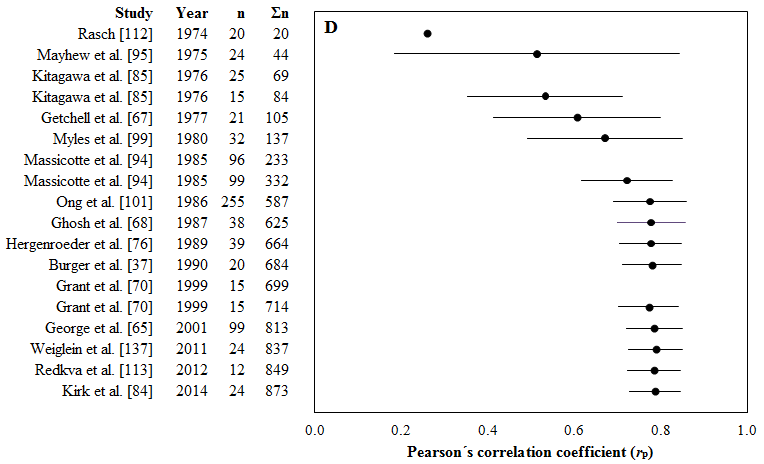


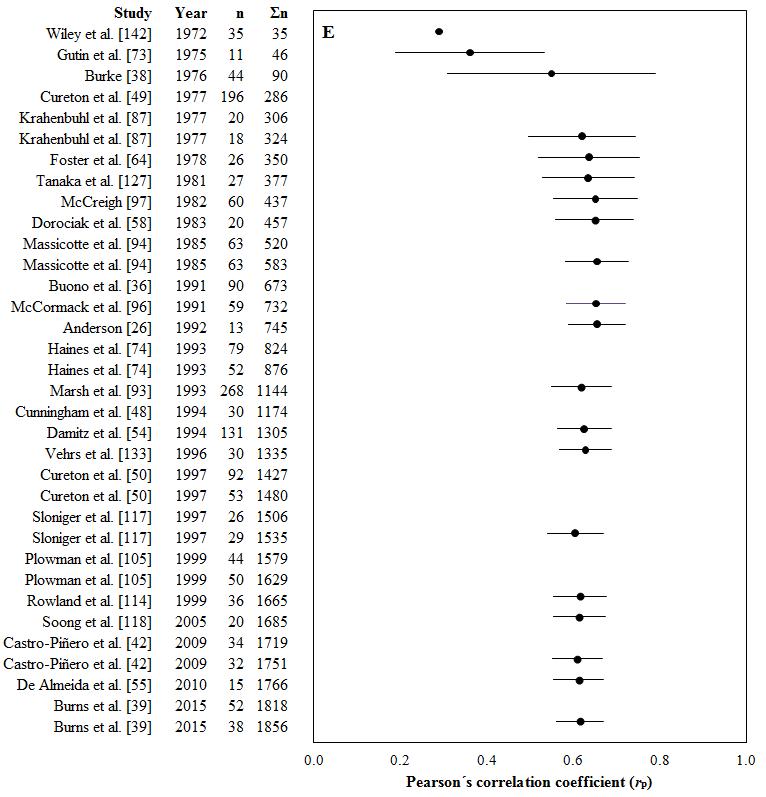


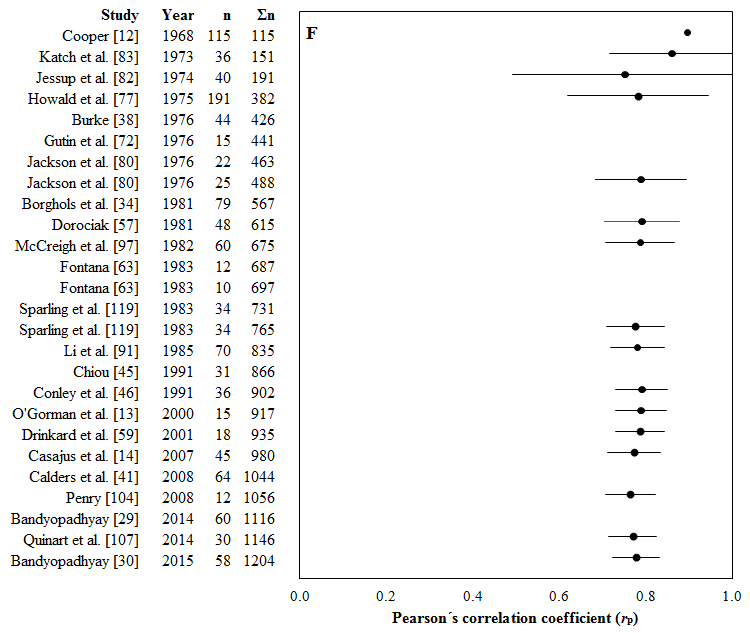


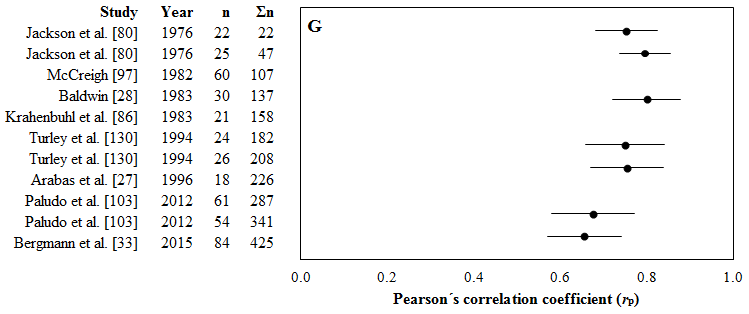

Supplement: S1 Fig — (DOC) [file pone.0151671.s002.doc]
