# Supplementary material for: Criterion-Related Validity of the Distance- and Time-Based Walk/Run Field Tests for Estimating Cardiorespiratory Fitness: A Systematic Review and Meta-Analysis
Source: PLoS One. 2016 Mar 17;11(3):e0151671. doi: 10.1371/journal.pone.0151671 (PMC4795745; doi:10.1371/journal.pone.0151671)
Supplement: S1 Table — (DOC) [file pone.0151671.s003.doc]

**S1 Table. Summary of the included studies examining the criterion-related validity of walk/run field tests for estimating cardiorespiratory fitness**

| Source | *n* | | Sex | Age | Fitness | Field Test | Criterion Test | Validity^a^ |
| --- | --- | --- | --- | --- | --- | --- | --- | --- |
| *Only performance score* | | | | | | | | |
| Barbineau et al. [31] | | 23 | Men | Adults | 64.92 | 5,000 m | Treadmill | 0.84 |
| Fay et al. [61] | | 13 | Women | Adults | 59.65 | 5,000 m | Treadmill | 0.91 |
| Guo-jun et al. [71] | | 63 | Men | Adults | 57.80 | 5,000 m | Treadmill | 0.32 |
| Ishiko [79] | | 17 | ? | Adults | 39.53 | 5,000 m | Cycle ergometer | 0.67 |
| Kumagai et al. [88] | | 12 | Men | Children | 64.10 | 5,000 m | Treadmill | 0.65 |
| Ramsbottom et al. [108] | | 36 | Men | Adults | 58.50 | 5,000 m | Treadmill | 0.76 |
| Ramsbottom et al. [108] | | 38 | Women | Adults | 47.40 | 5,000 m | Treadmill | 0.83 |
| Ramsbottom et al. [109] | | 69 | Men | Adults | 57.60 | 5,000 m | Treadmill | 0.85 |
| Ramsbottom et al. [109] | | 55 | Women | Adults | 46.60 | 5,000 m | Treadmill | 0.80 |
| Ramsbottom et al. [110] | | 18 | Men | Adults | 60.60 | 5,000 m | Treadmill | 0.89 |
| Ramsbottom et al. [110] | | 13 | Women | Adults | 53.10 | 5,000 m | Treadmill | 0.92 |
| Ramsbottom et al. [111] | | 12 | Mixed | Adults | 56.45 | 5,000 m | Treadmill | 0.89 |
| Scott et al. [115] | | 14 | Men | Adults | 60.40 | 5,000 m | Treadmill | 0.80 |
| Stratton et al. [122] | | 39 | Mixed | Adults | 43.90 | 5,000 m | Treadmill | 0.55 |
| Sucec et al. [124] | | 20 | Men | Adults | 63.10 | 5,000 m | Treadmill | 0.53 |
| Sucec et al. [124] | | 20 | Women | Adults | 57.40 | 5,000 m | Treadmill | 0.80 |
| Tanaka et al. [128] | | 18 | Men | Adults | 43.80 | 5,000 m | Cycle ergometer | 0.77 |
| Tanaka et al. [129] | | 29 | Men | Adults | 47.30 | 5,000 m | Cycle ergometer | 0.82 |
| Weyand et al. [141] | | 27 | Mixed | Adults | 61.48 | 5,000 m | Treadmill | 0.93 |
| Epperson et al. [60] | | 15 | Women | Adults | 58.10 | 3 miles | Treadmill | 0.66 |
| Jackson et al. [81] | | 50 | Men | Adults | 54.23 | 3 miles | Treadmill | 0.58 |
| Myles et al. [99] | | 32 | Men | Adults | 48.00 | 3 miles | Treadmill | 0.83 |
| Rasch [112] | | 18 | Men | Adults | 45.80 | 3 miles | Treadmill | 0.37 |
| Tanaka et al. [127] | | 27 | Men | Mixed | 70.10 | 3 miles | Treadmill | 0.70 |
| Wiley et al. [142] | | 35 | Men | Adults | ? | 3 miles | Treadmill | 0.43 |
| Cisar et al. [44] | | 32 | Men | Adults | 55.46 | 2 miles | Treadmill | 0.82 |
| Dorociak et al. [58] | | 20 | Women | Adults | 41.20 | 2 miles | Treadmill | 0.67 |
| Foster et al. [64] | | 26 | ? | Adults | 60.90 | 2 miles | Treadmill | 0.87 |
| Gutin et al. [73] | | 11 | Men | Adults | 52.50 | 2 miles | Treadmill | 0.41 |
| Mello et al. [98] | | 44 | Men | Adults | 50.40 | 2 miles | Treadmill | 0.91 |
| Mello et al. [98] | | 17 | Women | Adults | 42.00 | 2 miles | Treadmill | 0.89 |
| Sporiš [120] | | 409 | Men | Adults | 50.29 | 2 miles | Treadmill | 0.64 |
| Sporiš et al. [121] | | 30 | Men | Adults | 60.50 | 2 miles | Treadmill | 0.35 |
| Tanaka et al. [127] | | 27 | Men | Mixed | 70.10 | 2 miles | Treadmill | 0.69 |
| Walker et al. [136] | | 199 | Men | Adults | 50.90 | 2 miles | Treadmill | 0.65 |
| Weltman et al. [138] | | 44 | Women | Adults | 42.50 | 2 miles | Treadmill | 0.94 |
| Weltman et al. [139] | | 28 | Women | Adults | 38.50 | 2 miles | Treadmill | 0.77 |
| Weltman et al. [140] | | 29 | Men | Adults | 64.80 | 2 miles | Treadmill | 0.73 |
| Wiley et al. [142] | | 35 | Men | Adults | ? | 2 miles | Treadmill | 0.47 |
| Brandon et al. [35] | | 56 | Men | Adults | 62.50 | 3,000 m | Treadmill | 0.79 |
| Da Silva et al. [52] | | 17 | Men | Adults | 69.36 | 3,000 m | Treadmill | 0.62 |
| Da Silva et al. [52] | | 15 | Women | Adults | 57.42 | 3,000 m | Treadmill | 0.50 |
| Grant et al. [69] | | 16 | Men | Adults | 73.30 | 3,000 m | Treadmill | 0.70 |
| Mahon et al. [92] | | 19 | Men | Children | 54.60 | 3,000 m | Treadmill | 0.61 |
| O'Gorman et al. [13] | | 15 | Men | Adults | 59.80 | 3,000 m | Treadmill | 0.67 |
| Slattery et al. [116] | | 16 | Men | Adults | 55.70 | 3,000 m | Treadmill | 0.80 |
| Sucec et al. [124] | | 20 | Men | Adults | 63.10 | 3,000 m | Treadmill | 0.52 |
| Sucec et al. [124] | | 20 | Women | Adults | 57.40 | 3,000 m | Treadmill | 0.75 |
| Suh et al. [125] | | 24 | Men | Adults | 50.56 | 3,000 m | Treadmill | 0.66 |
| Suh et al. [125] | | 21 | Women | Adults | 41.85 | 3,000 m | Treadmill | 0.89 |
| Unnithan et al. [131] | | 13 | Men | Children | 60.50 | 3,000 m | Treadmill | 0.83 |
| Burger et al. [37] | | 20 | Men | Adults | 60.43 | 1.5 mile | Treadmill | 0.88 |
| George et al. [65] | | 99 | Mixed | Adults | 45.80 | 1.5 mile | Treadmill | 0.86 |
| Getchell et al. [67] | | 21 | Women | Adults | 46.20 | 1.5 mile | Treadmill | 0.92 |
| Ghosh et al. [68] | | 38 | Men | Adults | 55.96 | 1.5 mile | Cycle ergometer | 0.82 |
| Grant et al. [70] | | 15 | Men | Adults | 50.00 | 1.5 mile | Treadmill | 0.41 |
| Grant et al. [70] | | 15 | Women | Adults | 45.00 | 1.5 mile | Treadmill | 0.86 |
| Hergenroeder et al. [76] | | 39 | Mixed | Children | 49.09 | 1.5 mile | Cycle ergometer | 0.75 |
| Kirk et al. [84] | | 24 | Men | Adults | ? | 1.5 mile | Treadmill | 0.89 |
| Kitagawa et al. [85] | | 25 | Men | Adults | 51.80 | 1.5 mile | Treadmill | 0.63 |
| Kitagawa et al. [85] | | 15 | Women | Adults | 39.20 | 1.5 mile | Treadmill | 0.46 |
| Massicotte et al. [94] | | 96 | Men | Children | 56.34 | 1.5 mile | Cycle ergometer | 0.72 |
| Massicotte et al. [94] | | 99 | Women | Children | 45.20 | 1.5 mile | Cycle ergometer | 0.77 |
| Mayhew et al. [95] | | 24 | Men | Adults | 55.50 | 1.5 mile | Treadmill | 0.74 |
| Myles et al. [99] | | 32 | Men | Adults | 48.00 | 1.5 mile | Treadmill | 0.88 |
| Ong et al. [101] | | 255 | Men | Adults | 41.90 | 1.5 mile | Treadmill | 0.82 |
| Rasch [112] | | 20 | Men | Adults | 45.80 | 1.5 mile | Treadmill | 0.26 |
| Redkva et al. [113] | | 12 | Men | Adults | 57.96 | 1.5 mile | Treadmill | 0.61 |
| Weiglein et al. [137] | | 24 | Men | Adults | 50.30 | 1.5 mile | Treadmill | 0.89 |
| Anderson [26] | | 13 | Men | Children | 41.50 | 1 mile | Cycle ergometer | 0.83 |
| Buono et al. [36] | | 90 | Mixed | Children | 47.80 | 1 mile | Treadmill | 0.73 |
| Burke [38] | | 44 | Men | Adults | 52.79 | 1 mile | Treadmill | 0.74 |
| Burns et al. [39] | | 52 | Men | Children | 49.26 | 1 mile | Treadmill | 0.72 |
| Burns et al. [39] | | 38 | Women | Children | 39.95 | 1 mile | Treadmill | 0.62 |
| Castro-Piñero et al. [42] | | 34 | Men | Children | 61.00 | 1 mile | Treadmill | 0.50 |
| Castro-Piñero et al. [42] | | 32 | Women | Children | 53.00 | 1 mile | Treadmill | 0.54 |
| Cunningham et al. [48] | | 30 | Mixed | Children | 58.20 | 1 mile | Treadmill | 0.56 |
| Cureton et al. [49] | | 196 | Mixed | Children | 47.27 | 1 mile | Treadmill | 0.66 |
| Cureton et al. [50] | | 92 | Men | Children | 53.10 | 1 mile | Treadmill | 0.27 |
| Cureton et al. [50] | | 53 | Women | Children | 45.80 | 1 mile | Treadmill | 0.38 |
| Damitz et al. [54] | | 131 | Mixed | Children | 47.33 | 1 mile | Treadmill | 0.68 |
| De Almeida et al. [55] | | 15 | Men | Adults | 49.90 | 1 mile | Treadmill | 0.96 |
| Dorociak et al. [58] | | 20 | Women | Adults | 41.20 | 1 mile | Treadmill | 0.62 |
| Foster et al. [64] | | 26 | ? | Adults | 60.90 | 1 mile | Treadmill | 0.84 |
| Gutin et al. [73] | | 11 | Men | Adults | 52.50 | 1 mile | Treadmill | 0.63 |
| Haines et al. [74] | | 79 | Men | Children | 53.90 | 1 mile | Treadmill | 0.59 |
| Haines et al. [74] | | 52 | Women | Children | 45.20 | 1 mile | Treadmill | 0.13 |
| Krahenbuhl et al. [87] | | 20 | Men | Children | 47.60 | 1 mile | Treadmill | 0.71 |
| Krahenbuhl et al. [87] | | 18 | Women | Children | 42.90 | 1 mile | Treadmill | 0.26 |
| Marsh et al. [93] | | 268 | Mixed | Children | 49.54 | 1 mile | Treadmill | 0.60 |
| Massicotte et al. [94] | | 63 | Men | Children | 54.26 | 1 mile | Cycle ergometer | 0.66 |
| Massicotte et al. [94] | | 63 | Women | Children | 47.10 | 1 mile | Cycle ergometer | 0.67 |
| McCormack et al. [96] | | 59 | Mixed | Children | 50.84 | 1 mile | Treadmill | 0.48 |
| McCreigh [97] | | 60 | Women | Children | 48.58 | 1 mile | Treadmill | 0.75 |
| Plowman et al. [105] | | 44 | Men | Adults | 53.54 | 1 mile | Treadmill | 0.69 |
| Plowman et al. [105] | | 50 | Women | Adults | 47.41 | 1 mile | Treadmill | 0.79 |
| Rowland et al. [114] | | 36 | Men | Children | 47.00 | 1 mile | Cycle ergometer | 0.77 |
| Sloniger et al. [117] | | 26 | Men | Adults | 56.60 | 1 mile | Treadmill | 0.81 |
| Sloniger et al. [117] | | 29 | Women | Adults | 45.90 | 1 mile | Treadmill | 0.75 |
| Soong et al. [118] | | 20 | Mixed | Children | ? | 1 mile | Treadmill | 0.40 |
| Tanaka et al. [127] | | 27 | Men | Mixed | 70.10 | 1 mile | Treadmill | 0.62 |
| Vehrs et al. [133] | | 30 | Mixed | Adults | 45.05 | 1 mile | Treadmill | 0.75 |
| Wiley et al. [142] | | 35 | Men | Adults | ? | 1 mile | Treadmill | 0.29 |
| Díaz et al. [56] | | 51 | Mixed | Adults | 43.70 | 1,000 m | Treadmill | 0.86 |
| Rasch [112] | | 20 | Men | Adults | 45.80 | 1,000 m | Treadmill | 0.42 |
| Almarwaey et al. [25] | | 18 | Men | Children | 65.20 | ½ mile | Treadmill | 0.30 |
| Almarwaey et al. [25] | | 14 | Women | Children | 56.60 | ½ mile | Treadmill | 0.18 |
| Brandon et al. [35] | | 56 | Men | Adults | 62.50 | ½ mile | Treadmill | 0.66 |
| Castro-Piñero et al. [43] | | 47 | Mixed | Children | 72.40 | ½ mile | Treadmill | 0.55 |
| Castro-Piñero et al. [43] | | 39 | Mixed | Children | 61.60 | ½ mile | Treadmill | 0.53 |
| Gutin et al. [73] | | 11 | Men | Adults | 65.00 | ½ mile | Treadmill | 0.29 |
| Ingham et al. [78] | | 15 | Men | Adults | 61.48 | ½ mile | Treadmill | 0.75 |
| Ingham et al. [78] | | 16 | Women | Adults | 41.80 | ½ mile | Cycle ergometer | 0.74 |
| Yoshida et al. [143] | | 25 | Men | Children | 41.80 | ½ mile | Cycle ergometer | 0.57 |
| Ferfila et al. [62] | | 28 | Mixed | Children | 46.90 | 600 m | Treadmill | 0.54 |
| Burke et al. [38] | | 44 | Men | Adults | 52.79 | 600 yd | Treadmill | 0.78 |
| Cureton et al. [49] | | 196 | Mixed | Children | 47.27 | 600 yd | Treadmill | 0.62 |
| Gutin et al. [72] | | 15 | Mixed | Children | 47.50 | 600 yd | Treadmill | 0.71 |
| Hamlin et al. [75] | | 25 | Men | Children | 49.30 | 600 yd | Treadmill | 0.67 |
| Hamlin et al. [75] | | 28 | Women | Children | 46.10 | 600 yd | Treadmill | 0.50 |
| Krahenbuhl et al. [87] | | 20 | Men | Children | 47.60 | 600 yd | Treadmill | 0.58 |
| Krahenbuhl et al. [87] | | 18 | Women | Children | 42.90 | 600 yd | Treadmill | 0.03 |
| Vodak et al. [134] | | 69 | Men | Children | 53.60 | 600 yd | Treadmill | 0.50 |
| Chiou [45] | | 31 | ? | Adults | ? | ¼ mile | Treadmill | 0.88 |
| Dal Pupo et al. [53] | | 14 | Men | Adults | 64.17 | ¼ mile | Treadmill | 0.49 |
| Gutin et al. [73] | | 11 | Men | Adults | 52.50 | ¼ mile | Treadmill | 0.08 |
| Nevill et al. [100] | | 14 | Mixed | Adults | 65.00 | ¼ mile | Treadmill | 0.78 |
| Ruiz et al. [10] | | 43 | Mixed | Children | 56.70 | ¼ mile | Treadmill | 0.65 |
| Ruiz et al. [10] | | 43 | Mixed | Children | 57.70 | ¼ mile | Treadmill | 0.48 |
| Weyand et al. [141] | | 41 | Mixed | Adults | 61.48 | ¼ mile | Treadmill | 0.08 |
| Wiley et al. [142] | | 35 | Men | Adults | ? | ¼ mile | Treadmill | 0.22 |
| Pomerants [106] | | 102 | Women | Children | 39.35 | 15 min | Treadmill | 0.35 |
| Sucec et al. [123] | | 18 | Men | Adults | ? | 15 min | Treadmill | 0.81 |
| Bandyopadhyay [29] | | 60 | Women | Adults | 32.80 | 12 min | Cycle ergometer | 0.88 |
| Bandyopadhyay [30] | | 58 | Men | Adults | 39.80 | 12 min | Cycle ergometer | 0.93 |
| Borghols et al. [34] | | 79 | Men | Adults | 48.00 | 12 min | Treadmill | 0.73 |
| Burke [38] | | 44 | Men | Adults | 52.79 | 12 min | Treadmill | 0.90 |
| Calders et al. [41] | | 64 | Mixed | Children | 24.60 | 12 min | Cycle ergometer | 0.63 |
| Casajus et al. [14] | | 45 | Men | Adults | 54.90 | 12 min | Treadmill | 0.46 |
| Chiou [45] | | 31 | ? | Adults | ? | 12 min | Treadmill | 0.96 |
| Conley et al. [46] | | 36 | Men | Adults | 57.16 | 12 min | Treadmill | 0.88 |
| Cooper [12] | | 115 | Men | Adults | ? | 12 min | Treadmill | 0.90 |
| Dorociak [57] | | 48 | Women | Adults | 53.19 | 12 min | Treadmill | 0.89 |
| Drinkard et al. [59] | | 18 | Mixed | Children | 17.40 | 12 min | Cycle ergometer | 0.72 |
| Fontana [63] | | 12 | Men | Children | 56.94 | 12 min | Treadmill | 0.94 |
| Fontana [63] | | 10 | Women | Adults | 47.06 | 12 min | Treadmill | 0.73 |
| Gutin et al. [72] | | 15 | Mixed | Children | 47.50 | 12 min | Treadmill | 0.75 |
| Howald et al. [77] | | 191 | Men | Mixed | 55.81 | 12 min | Treadmill | 0.79 |
| Jackson et al. [80] | | 22 | Men | Children | 44.50 | 12 min | Treadmill | 0.82 |
| Jackson et al. [80] | | 25 | Women | Children | 40.62 | 12 min | Treadmill | 0.71 |
| Jessup et al. [82] | | 40 | Men | Adults | 48.46 | 12 min | Treadmill | 0.34 |
| Katch et al. [83] | | 36 | Women | Adults | 38.90 | 12 min | Treadmill | 0.67 |
| Li et al. [91] | | 70 | Men | Adults | 49.05 | 12 min | Treadmill | 0.82 |
| McCreigh et al. [97] | | 60 | Women | Children | 48.58 | 12 min | Treadmill | 0.73 |
| O'Gorman et al. [13] | | 15 | Men | Adults | 59.80 | 12 min | Treadmill | 0.67 |
| Penry [104] | | 12 | Women | Adults | 46.70 | 12 min | Treadmill | 0.89 |
| Quinart et al. [107] | | 30 | Mixed | Children | 23.70 | 12 min | Cycle ergometer | 0.70 |
| Sparling et al. [119] | | 34 | Men | Adults | 61.00 | 12 min | Treadmill | 0.69 |
| Sparling et al. [119] | | 34 | Women | Adults | 51.90 | 12 min | Treadmill | 0.62 |
| Arabas et al. [27] | | 18 | Men | Adults | 51.00 | 9 min | Treadmill | 0.83 |
| Baldwin [28] | | 30 | Men | Children | 51.80 | 9 min | Treadmill | 0.69 |
| Bergmann et al. [33] | | 84 | Mixed | Children | 44.40 | 9 min | Treadmill | 0.56 |
| Jackson et al. [80] | | 22 | Men | Children | 44.50 | 9 min | Treadmill | 0.82 |
| Jackson et al. [80] | | 25 | Women | Children | 40.62 | 9 min | Treadmill | 0.71 |
| Krahenbuhl et al. [86] | | 21 | Men | Children | 51.70 | 9 min | Treadmill | 0.99 |
| McCreigh [97] | | 60 | Women | Children | 48.58 | 9 min | Treadmill | 0.82 |
| Paludo et al. [103] | | 61 | Men | Children | 50.50 | 9 min | Treadmill | 0.59 |
| Paludo et al. [103] | | 54 | Women | Children | 42.31 | 9 min | Treadmill | 0.43 |
| Turley et al. [130] | | 24 | Men | Children | 41.20 | 9 min | Cycle ergometer | 0.68 |
| Turley et al. [130] | | 26 | Women | Children | 38.90 | 9 min | Cycle ergometer | 0.52 |
| Bergmann et al. [32] | | 125 | Mixed | Children | 44.49 | 6 min | Treadmill | 0.54 |
| Lawrenz et al. [90] | | 31 | Mixed | Children | 41.30 | 6 min | Treadmill | 0.46 |
| Swisher et al. [126] | | 30 | Mixed | Adults | 30.30 | 6 min | Treadmill | 0.79 |
| Van Mechelen et al. [132] | | 41 | Men | Children | 53.20 | 6 min | Treadmill | 0.51 |
| Van Mechelen et al. [132] | | 41 | Women | Children | 44.10 | 6 min | Treadmill | 0.45 |
| Vodak et al. [134] | | 69 | Men | Children | 53.60 | 6 min | Treadmill | 0.50 |
| Von Haaren et al. [135] | | 16 | Men | Children | 50.50 | 6 min | Treadmill | 0.80 |
| Von Haaren et al. [135] | | 14 | Women | Children | 49.20 | 6 min | Treadmill | 0.68 |
| *Performance score with other variables* | | | | | | | | |
| Mello et al. [98] | | 44 | Men | Adults | 50.40 | 2 miles | Treadmill | 0.94 |
| George et al. [66] | | 49 | Mixed | Adults | 48.10 | 1.5 mile | Treadmill | 0.90 |
| Getchell et al. [67] | | 21 | Women | Adults | 46.20 | 1.5 mile | Treadmill | 0.97 |
| Hergenroeder et al. [76] | | 39 | Mixed | Children | 49.09 | 1.5 mile | Cycle ergometer | 0.80 |
| Larsen et al. [89] | | 101 | Mixed | Adults | 46.00 | 1.5 mile | Treadmill | 0.90 |
| Buono et al. [36] | | 90 | Mixed | Children | 47.80 | 1 mile | Treadmill | 0.84 |
| Burns et al. [40] | | 90 | Mixed | Children | 45.23 | 1 mile | Treadmill | 0.81 |
| Cureton et al. [51] | | 753 | Mixed | Mixed | 48.37 | 1 mile | Treadmill | 0.72 |
| Damitz et al. [54] | | 131 | Mixed | Children | 47.33 | 1 mile | Treadmill | 0.82 |
| George et al. [66] | | 54 | Mixed | Adults | 46.60 | 1 mile | Treadmill | 0.87 |
| Krahenbuhl et al. [87] | | 38 | Mixed | Children | 45.37 | 1 mile | Treadmill | 0.99 |
| Castro-Piñero et al. [43] | | 47 | Mixed | Children | 57.40 | ½ mile | Treadmill | 0.66 |
| Hamlin et al. [75] | | 53 | Mixed | Children | 47.60 | 600 yd | Treadmill | 0.65 |
| Borghols et al. [34] | | 79 | Men | Adults | 48.00 | 12 mini | Treadmill | 0.80 |
| Coolbaugh et al. [47] | | 50 | Mixed | Adults | 50.92 | 12 mini | Treadmill | 0.88 |
| Jessup et al. [82] | | 40 | Men | Adults | 48.46 | 12 mini | Treadmill | 0.69 |
| Baldwin et al. [28] | | 30 | Men | Children | 51.80 | 9 min | Treadmill | 0.81 |
| Bergmann et al. [33] | | 56 | Mixed | Children | 44.55 | 9 min | Treadmill | 0.73 |
| McCreigh [97] | | 60 | Women | Children | 48.58 | 9 min | Treadmill | 0.86 |
| Paludo et al. [102] | | 72 | Men | Children | 39.32 | 9 min | Treadmill | 0.71 |
| Paludo et al. [102] | | 65 | Women | Children | 39.32 | 9 min | Treadmill | 0.79 |
| Bergmann et al. [32] | | 87 | Mixed | Children | 44.95 | 6 min | Treadmill | 0.77 |

*Note*. This table shows all the studies included in the meta-analysis with the maximal oxygen uptake relative to body mass only. ? = no information; ^a^ Criterion-related validity values are reported in absolute values and in Pearson´s *r* correlation coefficients; in case of multiple values only the correlation coefficients used in the meta-analysis is reported.
